# Risk assessment for canine periodontal disease using a hybrid causal Bayesian network

**DOI:** 10.3389/fvets.2026.1781228

**Published:** 2026-04-23

**Authors:** Ciaran O’Flynn, Harriet Wright, Abigail O’Rourke, Anna Harding, Tom Williams, Corrin Wallis, Colin Harvey, Marika Constantaras, Norman Fenton

**Affiliations:** 1 Waltham Petcare Science Institute, Leicestershire, United Kingdom; 2 Machine Intelligence and Decision Systems Research Group, Queen Mary University of London, London, United Kingdom; 3 Charter Vets, Barnstaple, United Kingdom; 4 Colin Harvey LLC, Cherry Hill, NJ, United States; 5 Pet Specialists of Hawaii, Waipahu, HI, United States; 6 Agena Ltd, Cambridge, United Kingdom

**Keywords:** Bayesian network, clinical decision support, dentistry, periodontal disease, risk assessment

## Abstract

Periodontal disease is among the most common diagnoses in canine primary care yet remains significantly underdiagnosed. The disconnect between prevalence and detection represents a critical gap in veterinary preventive medicine. Disease risk depends on non-modifiable factors (breed, age, morphology) and modifiable factors (dental hygiene, professional care), yet evidence-based interventions remain underutilized. We developed and validated a hybrid Bayesian network for canine periodontal disease risk assessment that integrates multiple factors to quantify disease probability. A first directed acyclic graph (DAG) for periodontal disease was constructed to define and map causal relationships between risk factors. This was followed by the construction of a Bayesian network that integrated data from 9.5 million electronic health records, 2,600 owner questionnaires, previous studies and expert elicitation. The final network comprised 19 nodes with 101 states and over 33,200 conditional probabilities. The model successfully differentiated high-risk from low-risk breeds and captured associations with age, size, head shape and dental hygiene practices. Key clinical indicators showed strong predictive value: a prior periodontitis probability was 12.4%, which increased to 17.6% with biofilm presence, 24.0% with poor dental conformation and 47.0% with gingivitis. The network demonstrated robust performance across four independent validation datasets, with ROC AUC values ranging from 0.583 to 0.962, sensitivity from 0.639 to 0.913 and specificity from 0.300 to 0.906. This hybrid Bayesian network integrated diverse data sources whilst accounting for complex interactions between morphological, clinical and preventive factors. The model’s bidirectional inference enables risk calculation using any combination of the 19 nodes and can operate as both a probabilistic inference tool (capturing observed associations) and causal inference tool (predicting intervention outcomes). This approach provides a framework to support clinical decision-making and demonstrates the utility of hybrid Bayesian networks for complex veterinary conditions where traditional epidemiological approaches face limitations.

## 1 Introduction

Periodontal disease represents one of the most prevalent health conditions in companion animals, affecting 44–100% of dogs (1). Despite its high prevalence, the condition remains significantly underdiagnosed in primary care settings (2) resulting in many dogs suffering from progressive oral pathology without clinical support (3–5). Canine periodontal disease encompasses two distinct conditions: gingivitis and periodontitis. Gingivitis is characterized by inflammation of gingival tissues and represents the initial reversible stage of disease. Without intervention, gingivitis can progress to periodontitis, where supporting tissues including the gingiva, alveolar bone, periodontal ligament, and cementum are irreversibly damaged (6). Importantly, gingivitis indicates active inflammation, whereas periodontitis represents the consequences and damage derived from previous episodes of disease where inflammation may no longer be present. The disconnect between disease burden and clinical recognition has profound implications, as untreated periodontal disease is progressive and can extend harm beyond tooth loss, with reported associations of periodontal disease in distant organs and to overall systemic health (3, 5, 7). Understanding the risk factors that influence the onset and progression of disease is therefore crucial for developing effective prevention strategies.

The contradiction of high prevalence alongside widespread underdiagnosis points to fundamental gaps in both awareness and intervention. The high prevalence is an avoidable burden, as periodontal disease is theoretically preventable in many cases (8, 9). Pet owners have access to numerous evidence-based preventative measures, including daily tooth brushing, dental chews, specialized dental diets, and professional dental care (10), however these remain severely underutilized. Likely due to the apparent burden of care (11), cost barriers; particularly for professional treatment (12, 13), and crucially, inadequate understanding of disease risk (14). The net effect is that many dogs fail to receive consistent preventative care, leading to disease development.

The underdiagnosis of periodontal disease reflects additional complexity. Whilst veterinarians typically understand the disease etiology, involving both modifiable and non-modifiable risk factors, and can identify at-risk patients, definitive diagnosis requires clinical examination and often general anesthesia and radiographic imaging (15). These diagnostic procedures depend on owner initiative and consent. Limited owner awareness compounds this challenge, as oral health checks that could both prevent disease progression and enable early detection are not prioritized (11, 14). Creating a cycle: where lack of awareness leads to delayed presentation, resulting in underdiagnosis, more advanced disease at diagnosis, and missed opportunities for prevention. This is a pattern unfortunately observed across many healthcare domains (16).

Multiple non-modifiable factors significantly influence periodontal disease risk in dogs. Breed predisposition is well-documented, with poodles, spaniels, terriers and greyhounds frequently observed at elevated risk (1, 2, 17). Body size demonstrates a strong inverse relationship with disease risk: small dogs face increased susceptibility, likely due to proportionally larger teeth resulting in poor dental conformations such as malocclusion and overcrowding, which create additional sites for plaque accumulation (4, 8). Skull morphology further compounds risk, with brachycephalic breeds reported to have 1.25 times increased odds of developing periodontal disease compared with mesocephalic types (2). Age is one of the strongest disease risk predictors (18–20), with incidence increasing progressively: dogs aged 12 and over are almost four times more likely to have periodontal disease than those aged 2–4 years (2). Though the causal relationships remains unclear, associations between age and periodontitis may reflect multiple mechanisms including the cumulative nature of periodontitis where destruction of the periodontium progresses over time (8), immune function decline, oral microbiome changes, and potentially increased diagnostic scrutiny in older dogs (21–23).

In contrast, modifiable factors present opportunities for risk reduction. Prevention strategies aim to establish and maintain health by reducing plaque accumulation and the subsequent inflammatory response (8). The recommended standard of care combines regular veterinary-led scale and polish procedures with daily owner-led tooth brushing (15), with studies identifying increased disease likelihood as time since professional cleaning extends (17). Other risk reduction factors include the use of specialized dental diets, dental chews and other science backed products (15). Modifiable and non-modifiable risk factors have interactions; tooth brushing effectiveness and compliance may vary between breeds due to anatomical differences, behavioral traits, or outcomes from interventions may be influenced by the age they are first applied (24). Tailoring preventive strategies to specific risk profiles could optimize both clinical outcomes and resource prioritization.

Whilst clinical trials remain the expected standard for establishing causality, their application to periodontal disease prevention faces significant constraints. Typically, studies focus on small cohorts of specific breeds or life stages (18–20, 25–27) limiting generalizability across diverse populations. Intervention studies examining homecare predominantly follow ‘clean mouth’ protocols (28–30), yet many dogs lack regular professional treatment, making it difficult to assess preventive effects in real-world populations. Furthermore, periodontal disease progresses through active and inactive stages, with periodontitis developing over years (19, 31), whilst practical and financial constraints limit most studies to several months.

Given these constraints, observational studies offer a valuable complementary approach. Analysis of real-world data from large electronic medical record datasets have identified numerous risk factors including bodyweight, age, weight status, and skull conformation (2, 17). However, epidemiological studies consistently report lower prevalence estimates compared to prospective studies (10–20% versus 50–100%) (1), suggesting substantial underdiagnosis. Data collected for clinical rather than research purposes vary in quality, with critical information about client behaviors, such as home care routines or diet types, often missing or incomplete. Establishing causal relationships poses additional challenges: the temporal sequence of cause and effect becomes difficult to determine when records are sparse, or event timing is unclear (32). These limitations highlight the need for methodological approaches that can augment incomplete data with clinical knowledge, whilst accounting for complex causal relationships.

Bayesian networks offer a solution to these challenges, providing a mathematical framework for representing complex probabilistic relationships (33–35). These graphical models use directed acyclic graphs (DAGs) to map causal pathways between variables, making assumptions transparent (36, 37). This framework supports two complementary analytical approaches: probabilistic inference, which quantifies statistical associations, and causal inference, which can employ Pearl’s do-calculus (34) to eliminate confounding variables and estimate the impact of interventions. Successfully applied to complex health conditions with multiple interacting factors and incomplete data (38–40), hybrid Bayesian networks can combine data-driven insights with expert knowledge within a single model. For periodontal disease, this approach takes insights from real-world data to understand risk across multiple breeds and life stages, whilst expert inputs address data gaps such as home care practices. Despite their structural complexity, these models can be communicated simply to both veterinarians and pet owners, providing an effective platform for risk assessment and clinical decision-making.

This study aims to develop and validate a hybrid causal Bayesian network for periodontal disease risk in dogs. The primary objective is to create a comprehensive model that integrates objective epidemiological data from primary care records with subjective knowledge from veterinary expertise to predict individual disease risk across canine populations. Secondary objectives include identifying key causal pathways influencing disease development, quantifying the relative importance of modifiable versus non-modifiable risk factors, and developing a communicable risk tool suitable for clinical practice. This work makes several novel contributions: it presents the first construction of a causal DAG for periodontal disease in dogs and demonstrates the first application of hybrid Bayesian networks to this condition, providing a platform for future studies and conversation.

## 2 Methods

### 2.1 Construction of the DAG

A directed acyclic graph (DAG) was constructed to identify risk factors associated with periodontal disease in dogs, combining literature review with domain expertise from veterinarians specializing in canine oral health. The DAG was developed through an iterative process to identify directional putative causal relationships between variables. Variables encompassed signalment factors (age, breed), preventive interventions (professional treatment, home care), diagnostic indicators, and clinical observations (Supplementary Table 1). Temporal considerations (e.g., the timing of observed clinical signs relative to disease diagnosis) were incorporated to ensure variable definitions aligned appropriately with downstream causal pathways.

### 2.2 Data selection

Following DAG construction, appropriate data sources were identified and relevant variables extracted. The study utilized observational real-world datasets comprising electronic health records (EHR) from general practice and pet owner questionnaires, referred to throughout as the ‘observational’ datasets. Variables were classified as either non-modifiable or modifiable. Non-modifiable variables (e.g., breed) remain constant throughout the animal’s lifetime. Modifiable variables included interventions (e.g., tooth brushing) and time-varying clinical states (e.g., gingivitis diagnosis).

#### 2.2.1 Electronic health records

Retrospective EHRs were extracted from dogs visiting Banfield® Pet Hospitals, a network of over 1,000 primary care veterinary hospitals in North America. Owners consented to anonymized data retention and use in research at registration. Further details have been previously described (17, 41). Extracted variables included signalment data (breed, mixed breed status, birth date) and visit-level data (visit type, reason, date, body condition score on a five-point scale, body weight in kg, billed items, diagnoses, and clinical signs). Dogs were required to have a minimum of two hospital visits for inclusion. One visit per dog was randomly selected as the outcome visit for periodontitis status evaluation. Variables from all records preceding this visit were used to populate node variable values (Supplementary Table 2). This dataset is referred to as the ‘EHR’ dataset.

#### 2.2.2 Questionnaires

Questionnaire data were obtained from pet owners registered with NomNomNow, Inc. (Nashville, TN, USA), a fresh pet food and health company (42). Owners completed questionnaires covering pet demographics and health. All participants provided consent for the use of their data in research. No personally identifiable information (PII) was collected. Variables included breed, birth date, date of questionnaire, periodontal disease status, concurrent diagnoses, clinical signs, oral care practices, and diet/treat preferences, behavioral factors, personality type, and time since veterinary visit. A minimum of two questionnaire responses at different timepoints were required for inclusion. The most recent questionnaire reporting periodontal disease status served as the outcome timepoint. Variables for the remaining DAG nodes were constructed from questionnaires completed prior to the outcome timepoint (Supplementary Table 2). Questionnaire responses were deemed valid for inclusion, even if optional questions were not completed. This dataset is referred to as the ‘questionnaire’ dataset.

#### 2.2.3 Other data sources

Data from previous studies were used as priors within the model and for the evaluation of the model. Demographic data was obtained from a meta-analysis aggregating records from over 4.3 million UK dogs across multiple sources, including EHRs, insurance databases, charity registries, and academic studies (43). Breed, head shape and age distributions from this dataset were used as priors in the model to increase generalizability. This dataset is subsequently referred to as the ‘demographic’ dataset. Data were extracted from a vignette-based survey of 462 veterinary practitioners who assessed periodontal disease risk using limited clinical information (44). The vignettes comprised dog attributes, oral hygiene regimens, and clinical signs. This dataset is subsequently referred to as the ‘expert’ dataset. An additional validation dataset comprised 127 client-owned dogs from a prospective study (45). Dogs scheduled for routine dental cleaning who underwent oral health assessments both before and during anesthesia. This dataset comprised demographic and clinical visit data from a balanced cohort with respect to signalment, but with high disease prevalence vs. EHR data; it is subsequently referred to as the ‘prospective’ dataset.

#### 2.2.4 Data processing

The same processing steps were applied to both EHR and questionnaire datasets using Python (v3.9) (46) with pyspark (v3.3.0) (47) and pandas (v1.3.5) (48) packages. Age at outcome was calculated as the interval between birth date and outcome date (randomly selected EHR or final questionnaire). Dogs younger than one month at outcome were excluded. Age was categorized into 15 bins as: <6 months, ≥6 months to <1 year, one-year intervals between ≥1 and < 13, and ≥13 years.

A simplified breed list was created, based on popularity and prevalence to reduce modeling complexity. Prevalence of periodontitis at 3 years old was determined within the EHR data for each breed which had at least 1,000 records. The top 25 breeds, and any breeds more than one standard deviation away from the population mean, were selected. There was overlap between popular breeds and those showing deviation, resulting in 14 breeds with a higher prevalence, 15 breeds with a lower prevalence than the population average, and an additional 14 of the most common breeds. Breeds that fell outside of this selection were grouped as “Other.”

Head shape (brachycephalic, dolichocephalic, or mesocephalic) was assigned to each breed using an internal reference (Supplementary Table 4), supplemented by previously published classifications (2, 49). Breed size was determined using bodyweight data from dogs aged 2–10 years with ideal body condition scores in the EHR database (January 2015–March 2024). For breeds with ≥200 individuals and coefficient of variation ≤0.5 (indicating consistent within-breed weights), mean breed weight determined size category: toy (≤6.5 kg), small (>6.5 – ≤ 9 kg), medium-small (>9 – ≤ 15 kg), medium-large (>15 – ≤ 30 kg), large (>30 – ≤ 40 kg), and giant (>40 kg) (41). Mixed breeds, unknown breeds, or breeds not meeting these criteria were assigned size categories using the same thresholds based on average individual bodyweight between 2 and 10 years old, adjusted for BCS using a 20% correction per point on the BCS scale (50). If bodyweight was not available between 2 and 10 years old for an individual, breed size was not assigned. Where breed size and head shape were not available or unable to be assigned, the records were retained but with missing data.

Several structurally complex nodes were simplified into ranked nodes, calculated by summing weighted contributions from parent nodes, with the resulting scores categorized into ordinal levels (e.g., low, medium, high risk). Both the weighting scheme and threshold values for categorization were established through consensus expert opinion (Supplementary Table 3).

Alongside flagging for a periodontitis diagnosis, additional nodes were included for EHR where the diagnosis method was known. Each dataset was split into a training and test set with a ratio of 80:20, balanced on periodontitis status.

#### 2.2.5 Expert elicitation

For variables not recorded in the data, conditional probability tables (CPT) were elicited from panels comprising four independent experts, identified on the basis of demonstrated expertise in veterinary dentistry, with current and practical clinical experience with canine patients. Participants received background information including project objectives, fundamentals of causality and probability, and the proposed DAG with node definitions. Workshops were conducted with individuals between November 2024 and April 2025. For practicality, variables with high cardinality (many values) were simplified. Breeds were categorized by panelists into periodontitis risk levels (low, medium, high), with the highest assigned value used when panelists disagreed. Age was grouped into life stages, with categories refined during workshops based on panelist consensus. Each CPT was completed by at least two panelists, who documented their reasoning and confidence levels. Individual responses were combined using the mean and were reviewed by all panelists.

### 2.3 Validation of the DAG

The expert-informed DAG was iteratively validated through conditional independence tests (51), statistical experiments that determine whether two variables are independent given the values of a third, using DAGitty (v0.3.4) (36) in R (v4.1.3) (52). These tests identify correlations in the data that are not implied by the DAG. For edges appearing significant, a final decision as to inclusion and direction of the edge in the DAG was made based on domain knowledge and comparison of the Bayesian information criterion (BIC) scores via the bnlearn package (v4.9.4) (53).

The DAG was validated against each observational dataset (EHR and questionnaire) independently. For each validation, only nodes with available data in the respective dataset were included, with causal relationships retained by connecting nodes. Due to computational constraints, a random sample of 900,000 EHRs were used; the complete questionnaire dataset was used. Categorical variables were encoded as ordinal: head shape by increasing muzzle length (brachycephalic < mesocephalic < dolichocephalic); breed size by increasing weight categories; ranked nodes as bad < medium < good; and breeds by age-adjusted periodontitis prevalence determined in the EHR data (low < medium < high).

### 2.4 Network building and inference

The Bayesian network was constructed using Python (v3.9) (46) and pgmpy (v0.1.26) (54), following a node-by-node approach based on the validated DAG. Each node was assigned a prior, which was updated with suitable data (when available) to determine the posterior. Priors were specified as non-informative (uniform) or informative (derived from expert opinion or data). When multiple data sources were available, they were combined to create composite priors. Data was used to update priors only when the following criteria were met: (a) complete data was available for the node and all parent nodes, and (b) data quality was acceptable; assessed by consensus across breadth, representativeness, and alignment with intended node definitions. Weightings were required to (1) create composite priors, (2) combine datasets, and (3) weight priors within the data. These values were determined based on exploratory hyperparameter optimization and domain knowledge, considering factors such as data quality or relevance of the literature.

For all analyses, the model was queried using probabilistic inference, providing the inputs as evidence to the Variable Elimination algorithm (54). This emulates the real-world application of the model to predict the probability of periodontitis for a dog, given a set of observed data points. For expectation analysis and reporting model results, the model was additionally queried using causal inference. In causal inference, the ‘do’ operator is used to provide inputs as interventions to the model (34, 54, 55). This removes the effect of any confounding variables, and the result can be interpreted as the inputs *causing* the output. Jensen-Shannon Divergence (JSD) (56) was calculated to understand effect size for both types of inference. Thresholds for interpreting JSD were defined based on exploration within the model predictions and comparison to Cramer’s V with > 0.2 considered ‘very strong’, > 0.1 ‘strong’, > 0.05 ‘moderate’, > 0 ‘weak’, and equal to 0 ‘no effect’ (57, 58).

### 2.5 Model evaluation

Model evaluation comprised four components: individual node predictive performance, outcome uncertainty, sensitivity analysis of input variables, and overall predictive performance (59).

Individual node predictive performance was evaluated by assessing how parent nodes predicted their child values (e.g., predicting head shape from breed and breed size) using the observational validation datasets. Predictive performance metrics varied by variable type: binary nodes used balanced accuracy and ROC AUC; multiclass nodes used balanced accuracy and one-vs-one ROC AUC; ordinal nodes used mean absolute distance from true class. Performance was calculated only for nodes with parents and where complete parent–child data existed within a single dataset. Outcome uncertainty was assessed using the posterior probability certainty index (PPCI) (59), which quantifies how certain the model is regarding outcomes. This metric was calculated for all validation datasets and reported as dataset averages.

Sensitivity analysis identified nodes exerting the largest influence on the outcome. Two approaches were employed - entropy reduction and expectation analysis. Entropy reduction quantified each input node’s influence on the outcome by measuring the decrease in outcome uncertainty when that input was known. Expectation analysis tested whether model relationships aligned with predefined domain knowledge; for example, ‘periodontitis probability should increase with age’. Discrepancies would indicate potential issues in structure learning, data quality, or prior specification.

Predictive performance was evaluated using four independent datasets representing different clinical scenarios, data accuracy levels, and variable availability. Each contained different subsets of variables with heterogeneous node definitions. Performance metrics included ROC AUC, sensitivity, specificity, and balanced accuracy.

The datasets comprised: ‘Observational’, 20% holdout sets from (1) EHR validation data and (2) questionnaire validation data, representing general populations with signalment and periodontitis diagnoses in real-world settings; (3) ‘Prospective’, a research cohort with comprehensive data collection including detailed oral examinations whilst under general anesthetic; (4) ‘Expert’, veterinary assessments of periodontitis probability from clinical vignettes, representing collective professional judgment on fictional scenarios (44).

## 3 Results

### 3.1 Construction of the DAG

**Figure 1:**
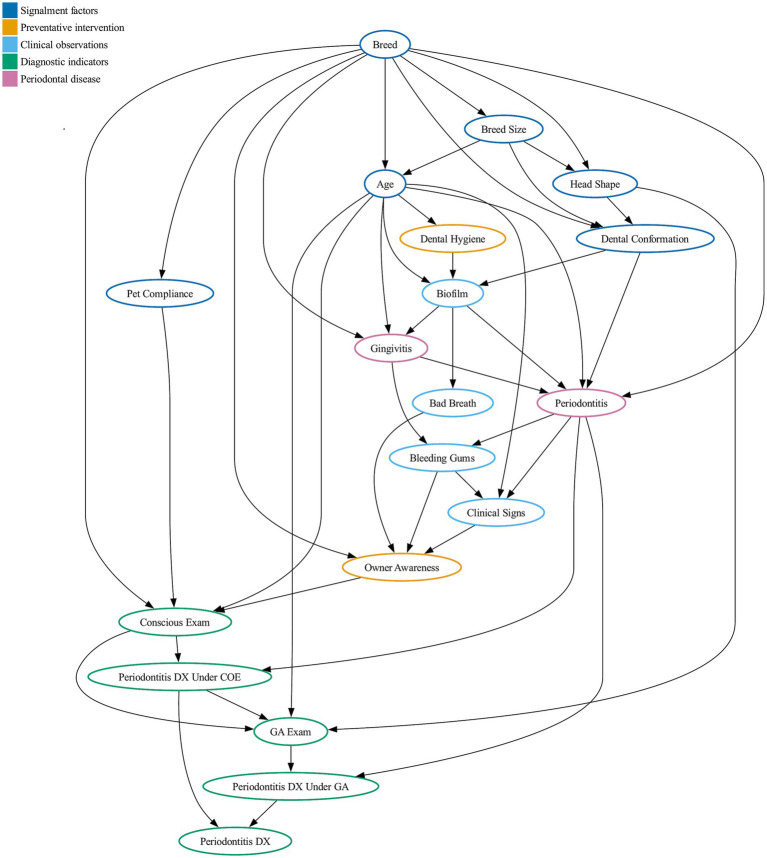
Directed acyclic graph showing hypothesized causal relationships of periodontal disease. Each node is representing a risk factor associated with periodontal disease, connected by edges representing directed causal relationships between the nodes, where conscious oral exam (COE), diagnosis (DX), and general anesthetic (GA).

The DAG development process produced a network (Figure 1) containing 19 nodes, 45 edges and 101 total states, requiring 33,231 conditional probabilities. Nodes represented four categories: pet attributes, dental hygiene practices, clinical signs, and disease-diagnosis relationships. Along with the construction of the DAG the development process also produced definitions for variables and interactions (Supplementary Table 1).

DAG validation against observational data identified two additional relationships: age affecting both dental hygiene and clinical signs. Validation also suggested an increased likelihood of biofilm presence with good dental hygiene, however given the clinical implausibility and the sparseness of dental hygiene data this was rejected.

### 3.2 Description of the data

#### 3.2.1 Observational data

EHR data from 9,546,672 dogs between February 2010 and June 2025 were analyzed. Periodontitis prevalence was 9.16%, whilst periodontal disease prevalence was 30.83%. The EHR dataset contained data for 13 of 19 DAG nodes. Records for bad breath and dental hygiene practices (including tooth brushing) were sparse. Head shape data was missing for 8.23% of dogs, and breed size for 5.50%. The three most common breeds were American Staffordshire Terrier (9.33%), Chihuahua (8.15%), and Labrador Retriever (8.00%), with mixed breed making up 6.71%. Dogs aged 0–6 months comprised the largest group (28.56%). Table 1 summarizes the complete EHR dataset.

**Table 1:** Summary of count and percentage for the electronic health record dataset stratified by life stage and node value.

Node	Value	Total	Puppy	Youth	Mature	Senior	Super senior
Bad breath	False	9,539,359 (99.92%)	3,931,607 (41.21%)	3,720,942 (39.01%)	1,367,326 (14.33%)	475,911 (4.99%)	43,573 (0.46%)
	True	7,313 (0.08%)	1870 (25.57%)	3,870 (52.91%)	984 (13.45%)	497 (6.80%)	92 (1.26%)
Biofilm	False	3,581,312 (37.51%)	2,889,179 (80.67%)	613,281 (17.12%)	61,035 (1.70%)	16,247 (0.45%)	1,570 (0.04%)
	True	5,965,360 (62.49%)	1,044,298 (17.51%)	3,111,531 (52.16%)	1,307,275 (21.91%)	460,161 (7.71%)	42,095 (0.71%)
Breed	Other	1,500,464 (15.72%)	532,330 (35.48%)	621,433 (41.42%)	249,359 (16.62%)	89,219 (5.95%)	8,123 (0.54%)
	American Staffordshire Terrier	890,931 (9.33%)	475,196 (53.34%)	306,422 (34.39%)	85,241 (9.57%)	22,753 (2.55%)	1,319 (0.15%)
	Chihuahua	778,431 (8.15%)	297,457 (38.21%)	287,094 (36.88%)	132,170 (16.98%)	55,474 (7.13%)	6,236 (0.80%)
	Labrador Retriever	763,336 (8.0%)	308,235 (40.38%)	304,215 (39.85%)	112,469 (14.73%)	36,191 (4.74%)	2,226 (0.29%)
	Mixed Breed	640,137 (6.71%)	245,776 (38.39%)	256,982 (40.14%)	97,018 (15.16%)	36,654 (5.73%)	3,707 (0.58%)
	Yorkshire Terrier	493,209 (5.17%)	197,414 (40.03%)	185,004 (37.51%)	76,831 (15.58%)	30,695 (6.22%)	3,265 (0.66%)
	Shih Tzu	480,685 (5.04%)	192,219 (39.99%)	181,633 (37.79%)	73,274 (15.24%)	30,053 (6.25%)	3,506 (0.73%)
	German Shepherd	471,358 (4.94%)	222,192 (47.14%)	180,907 (38.38%)	54,003 (11.46%)	13,618 (2.89%)	638 (0.14%)
	Poodle	461,202 (4.83%)	187,719 (40.70%)	188,916 (40.96%)	59,856 (12.98%)	22,133 (4.80%)	2,578 (0.56%)
	Maltese	310,963 (3.26%)	121,168 (38.97%)	116,741 (37.54%)	50,858 (16.36%)	19,980 (6.43%)	2,216 (0.71%)
Breed size	Toy	2,182,953 (22.87%)	771,457 (35.34%)	858,473 (39.33%)	379,628 (17.39%)	155,692 (7.13%)	17,703 (0.81%)
	Small	1,146,878 (12.01%)	383,079 (33.40%)	462,764 (40.35%)	205,565 (17.92%)	85,519 (7.46%)	9,951 (0.87%)
	Medium Small	980,676 (10.27%)	336,628 (34.33%)	420,229 (42.85%)	160,469 (16.36%)	57,920 (5.91%)	5,430 (0.55%)
	Medium Large	2,531,786 (26.52%)	1,067,870 (42.18%)	1,027,819 (40.60%)	333,267 (13.16%)	96,588 (3.82%)	6,242 (0.25%)
	Large	1,799,013 (18.84%)	754,323 (41.93%)	724,502 (40.27%)	245,159 (13.63%)	71,117 (3.95%)	3,912 (0.22%)
	Giant	380,238 (3.98%)	184,393 (48.49%)	147,322 (38.74%)	40,309 (10.60%)	7,970 (2.10%)	244 (0.06%)
	N/A	525,128 (5.5%)	435,727 (82.98%)	83,703 (15.94%)	3,913 (0.75%)	1,602 (0.31%)	183 (0.03%)
Clinical signs	Bad	769,965 (8.07%)	64,352 (8.36%)	245,033 (31.82%)	280,579 (36.44%)	161,255 (20.94%)	18,746 (2.43%)
	Medium	15,784 (0.17%)	3,815 (24.17%)	6,401 (40.55%)	3,670 (23.25%)	1724 (10.92%)	174 (1.10%)
	Good	8,760,923 (91.77%)	3,865,310 (44.12%)	3,473,378 (39.65%)	1,084,061 (12.37%)	313,429 (3.58%)	24,745 (0.28%)
Conscious exam	False	2,366,277 (24.79%)	1,135,118 (47.97%)	869,560 (36.75%)	260,171 (10.99%)	92,268 (3.90%)	9,160 (0.39%)
	True	7,180,395 (75.21%)	2,798,359 (38.97%)	2,855,252 (39.76%)	1,108,139 (15.43%)	384,140 (5.35%)	34,505 (0.48%)
Dental conformation	Bad	606,633 (6.35%)	261,306 (43.07%)	255,810 (42.17%)	66,926 (11.03%)	20,694 (3.41%)	1897 (0.31%)
	Medium	842,717 (8.83%)	264,797 (31.42%)	367,625 (43.62%)	153,096 (18.17%)	52,746 (6.26%)	4,453 (0.53%)
	Good	8,097,322 (84.82%)	3,407,374 (42.08%)	3,101,377 (38.30%)	1,148,288 (14.18%)	402,968 (4.98%)	37,315 (0.46%)
Dental hygiene	Bad	7,579,019 (79.39%)	3,714,746 (49.01%)	2,825,818 (37.28%)	772,564 (10.19%)	242,662 (3.20%)	23,229 (0.31%)
	Medium	54,396 (0.57%)	17,575 (32.31%)	27,243 (50.08%)	7,438 (13.67%)	1950 (3.58%)	190 (0.35%)
	Good	1,913,257 (20.04%)	201,156 (10.51%)	871,751 (45.56%)	588,308 (30.75%)	231,796 (12.12%)	20,246 (1.06%)
GA exam	False	7,872,929 (82.47%)	3,763,711 (47.81%)	2,984,311 (37.91%)	840,191 (10.67%)	260,009 (3.30%)	24,707 (0.31%)
	True	1,673,743 (17.53%)	169,766 (10.14%)	740,501 (44.24%)	528,119 (31.55%)	216,399 (12.93%)	18,958 (1.13%)
Gingivitis	False	7,477,241 (78.32%)	3,720,150 (49.75%)	2,823,176 (37.76%)	724,895 (9.69%)	193,884 (2.59%)	15,136 (0.20%)
	True	2,069,431 (21.68%)	213,327 (10.31%)	901,636 (43.57%)	643,415 (31.09%)	282,524 (13.65%)	28,529 (1.38%)
Head shape	Brachycephalic	1,457,229 (15.26%)	616,316 (42.29%)	569,693 (39.09%)	198,086 (13.59%)	67,137 (4.61%)	5,997 (0.41%)
	Dolichocephalic	617,085 (6.46%)	260,019 (42.14%)	241,581 (39.15%)	84,898 (13.76%)	28,051 (4.55%)	2,536 (0.41%)
	Mesocephalic	6,686,232 (70.04%)	2,759,224 (41.27%)	2,594,389 (38.80%)	964,875 (14.43%)	336,935 (5.04%)	30,809 (0.46%)
	N/A	786,126 (8.23%)	297,918 (37.9%)	319,149 (40.60%)	120,451 (15.32%)	44,285 (5.63%)	4,323 (0.55%)
Periodontitis DX	False	8,672,495 (90.84%)	3,877,504 (44.71%)	3,434,315 (39.6%)	1,041,344 (12.01%)	296,190 (3.42%)	23,142 (0.27%)
	True	874,177 (9.16%)	55,973 (6.40%)	290,497 (33.23%)	326,966 (37.40%)	180,218 (20.62%)	20,523 (2.35%)
Periodontitis DX under COE	False	8,678,052 (90.9%)	3,877,572 (44.68%)	3,435,770 (39.59%)	1,043,769 (12.03%)	297,637 (3.43%)	23,304 (0.27%)
	True	868,620 (9.1%)	55,905 (6.44%)	289,042 (33.28%)	324,541 (37.36%)	178,771 (20.58%)	20,361 (2.34%)
Periodontitis DX under GA	False	9,353,975 (97.98%)	3,922,627 (41.94%)	3,666,458 (39.2%)	1,292,751 (13.82%)	433,279 (4.63%)	38,860 (0.42%)
	True	192,697 (2.02%)	10,850 (5.63%)	58,354 (30.28%)	75,559 (39.21%)	43,129 (22.38%)	4,805 (2.49%)

Including only the top 10 breeds by frequency. Where diagnosis (DX), conscious oral exam (COE), general anesthetic (GA). For life stages, puppy (0 ≤ 1 years), youth (1 ≤ 5 years), mature (5 ≤ 9 years) senior (9 ≤ 13 years) and super senior (>13 + years).

Questionnaire data were available from 2,633 dogs whose owners completed two surveys between November 2018 and May 2023. Periodontal disease was self-reported by 13.71% of owners. The questionnaires captured data for 8 of 19 DAG nodes. The three most common breeds were Chihuahua (4.94%), Yorkshire Terrier (3.95%), and Shih Tzu (3.04%) with mixed breed making up 32.36%. Head shape and breed size could not be assigned for 38.36 and 36.65% of dogs respectively, primarily due to mixed breed status precluding assignment. Dogs aged one year were most common, comprising 12.42% of the population. Table 2 summarizes this dataset.

**Table 2:** Summary of count and percentage for the questionnaire dataset stratified by life stage and node value.

Node	Value	Total	Puppy	Youth	Mature	Senior	Super senior
Bad breath	False	1,603 (60.88%)	268 (16.72%)	644 (40.17%)	363 (22.65%)	237 (14.78%)	91 (5.68%)
	True	1,030 (39.12%)	45 (4.36%)	279 (27.06%)	270 (26.19%)	295 (28.61%)	141 (13.68%)
Bleeding gums	False	2,580 (97.99%)	312 (12.09%)	909 (35.23%)	617 (23.91%)	515 (19.96%)	227 (8.80%)
	True	53 (2.01%)	1 (1.85%)	14 (25.93%)	16 (29.63%)	17 (31.48%)	5 (9.26%)
Breed	Mixed Breed	852 (32.36%)	94 (11.03%)	319 (37.44%)	215 (25.23%)	164 (19.25%)	60 (7.04%)
	Other	500 (18.99%)	62 (12.40%)	156 (31.20%)	138 (27.60%)	104 (20.80%)	40 (8.00%)
	Chihuahua	130 (4.94%)	7 (5.38%)	22 (16.92%)	37 (28.46%)	34 (26.15%)	30 (23.08%)
	Yorkshire Terrier	104 (3.95%)	10 (9.62%)	35 (33.65%)	10 (9.62%)	30 (28.85%)	19 (18.27%)
	Shih Tzu	80 (3.04%)	6 (7.50%)	27 (33.75%)	17 (21.25%)	20 (25.00%)	10 (12.50%)
	French Bulldog	76 (2.89%)	16 (21.05%)	48 (63.16%)	8 (10.53%)	4 (5.26%)	0 (0.00%)
	Poodle	75 (2.85%)	10 (13.33%)	25 (33.33%)	15 (20.00%)	12 (16.00%)	13 (17.33%)
	Labrador Retriever	73 (2.77%)	7 (9.59%)	22 (30.14%)	21 (28.77%)	15 (20.55%)	8 (10.96%)
	Dachshund	69 (2.62%)	7 (10.14%)	16 (23.19%)	22 (31.88%)	14 (20.29%)	10 (14.49%)
	American Staffordshire Terrier	61 (2.32%)	3 (4.92%)	26 (42.62%)	15 (24.59%)	12 (19.67%)	5 (8.20%)
Breed size	Toy	479 (18.19%)	42 (8.77%)	131 (27.35%)	110 (22.96%)	123 (25.68%)	73 (15.24%)
	Small	306 (11.62%)	32 (10.46%)	83 (27.12%)	77 (25.16%)	72 (23.53%)	42 (13.73%)
	Medium Small	314 (11.93%)	54 (17.2%)	123 (39.17%)	75 (23.89%)	43 (13.69%)	19 (6.05%)
	Medium Large	300 (11.39%)	38 (12.67%)	119 (39.67%)	65 (21.67%)	57 (19.00%)	21 (7.00%)
	Large	234 (8.89%)	40 (17.09%)	95 (40.60%)	49 (20.94%)	41 (17.52%)	9 (3.85%)
	Giant	35 (1.33%)	8 (22.86%)	15 (42.86%)	9 (25.71%)	3 (8.57%)	0 (0.00%)
	N/A	965 (36.65%)	99 (10.26%)	357 (36.99%)	248 (25.7%)	193 (20.00%)	68 (7.05%)
Clinical signs	Medium	1,338 (50.82%)	201 (15.02%)	468 (34.98%)	291 (21.75%)	252 (18.83%)	126 (9.42%)
	Good	1,295 (49.18%)	112 (8.65%)	455 (35.14%)	342 (26.41%)	280 (21.62%)	106 (8.19%)
Dental hygiene	Bad	260 (9.87%)	36 (13.85%)	90 (34.62%)	60 (23.08%)	43 (16.54%)	31 (11.92%)
	Medium	492 (18.69%)	60 (12.20%)	202 (41.06%)	124 (25.20%)	83 (16.87%)	23 (4.67%)
	Good	1881 (71.44%)	217 (11.54%)	631 (33.55%)	449 (23.87%)	406 (21.58%)	178 (9.46%)
Head shape	Brachycephalic	336 (12.76%)	46 (13.69%)	141 (41.96%)	75 (22.32%)	52 (15.48%)	22 (6.55%)
	Dolichocephalic	161 (6.11%)	23 (14.29%)	53 (32.92%)	33 (20.50%)	34 (21.12%)	18 (11.18%)
	Mesocephalic	1,126 (42.76%)	135 (11.99%)	359 (31.88%)	260 (23.09%)	251 (22.29%)	121 (10.75%)
	N/A	1,010 (38.36%)	109 (10.79%)	370 (36.63%)	265 (26.24%)	195 (19.31%)	71 (7.03%)
Periodontitis DX	False	2,272 (86.29%)	313 (13.78%)	889 (39.13%)	544 (23.94%)	392 (17.25%)	134 (5.90%)
	True	361 (13.71%)	0 (0.00%)	34 (9.39%)	89 (24.59%)	140 (38.67%)	98 (27.07%)

Where diagnosis (Dx). For life stages, puppy (0 ≤ 1 years), youth (1 ≤ 5 years), mature (5 ≤ 9 years) senior (9 ≤ 13 years) and super senior (>13 + years).

#### 3.2.2 Expert elicitation

Expert elicitation was required for nine nodes lacking observational data due to absence from datasets, missing parent nodes, or insufficient quality. Four nodes were absent from both datasets: periodontitis, conscious exam, owner awareness, and pet compliance. Four dependent nodes could not be populated without these parents: bleeding gums, clinical signs, and both periodontitis diagnosis nodes (under general anesthetic [GA] and conscious oral exam [COE]) required the periodontitis node, whilst conscious exam required pet compliance and owner awareness. Dental hygiene, despite complete network coverage, required expert elicitation due to sparse data.

**Figure 2:**
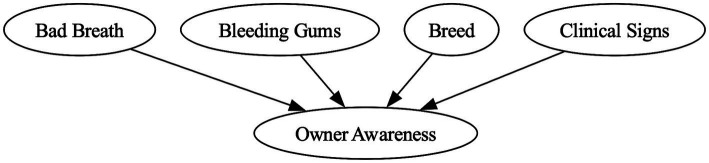
Directed acyclic graph illustrating hypothesized subgraph causal relationships of owner awareness showing parent nodes.

Expert elicitation produced a CPT for each targeted node, incorporating input from at least two experts. Table 3 shows an example CPT for owner awareness, with probability estimates based on clinical signs, breed risk category (defined in Supplementary Table 5), bleeding gums, and bad breath. Figure 2 shows the corresponding local DAG.

**Table 3:** Conditional probability table elicited from a panel of veterinarians with expertise in canine oral health, on the probability of the node “owner awareness” given the node parents, bad breath, bleeding gums, breed proxy and clinical signs (as defined in Supplementary Table 1), averaged across the expert panel.

Bad breath	Bleeding gums	Breed risk group	Clinical signs	P (owner awareness = False)	P (owner awareness = True)
False	False	Low	Bad	0.625	0.375
			Medium	0.523	0.478
			Good	0.970	0.030
		Expected	Bad	0.600	0.400
			Medium	0.473	0.528
			Good	0.945	0.055
		High	Bad	0.425	0.575
			Medium	0.375	0.625
			Good	0.750	0.250
	True	Low	Bad	0.525	0.475
			Medium	0.463	0.538
			Good	0.875	0.125
		Expected	Bad	0.500	0.500
			Medium	0.413	0.588
			Good	0.850	0.150
		High	Bad	0.275	0.725
			Medium	0.275	0.725
			Good	0.675	0.325
True	False	Low	Bad	0.525	0.475
			Medium	0.480	0.520
			Good	0.935	0.065
		Expected	Bad	0.500	0.500
			Medium	0.430	0.570
			Good	0.910	0.090
		High	Bad	0.300	0.700
			Medium	0.313	0.688
			Good	0.675	0.325
	True	Low	Bad	0.450	0.550
			Medium	0.388	0.613
			Good	0.800	0.200
		Expected	Bad	0.425	0.575
			Medium	0.343	0.658
			Good	0.775	0.225
		High	Bad	0.175	0.825
			Medium	0.193	0.808
			Good	0.600	0.400

### 3.3 Construction of the Bayesian network

The resulting discrete network comprised CPTs specifying probabilities for each node given its parent values. EHR data were used for 11 nodes, questionnaire data for 5 nodes, and data expert elicitation for 8 of the nodes. Table 4 details the priors, datasets, and the weights determined by parameter estimation used for each node.

**Table 4:** Model prior, data and weighting configuration for each node in the network.

Node name	Priors		Dataset	Weights		
	Other	Dataset		Priors	Datasets	Prior: Dataset
Age	D	EHR P (age | breed size)	Q, EHR	1:1	1:20	1:1
Bad breath			EHR sampled by Q P (bad breath)			
Biofilm			EHR sampled by E P (dental hygiene | biofilm)			
Bleeding gums	E					
Breed	D		Q, EHR		1:20	1:1
Breed size	D	M	Q, EHR	1:10	1:2	1:2
Clinical signs	E	Q P (clinical signs | bleeding gums) EHR P (clinical signs | age)		15:1:2		
Conscious exam	E	EHR P (conscious exam | age, breed)		5:1		
Dental conformation		M, EHR P (dental conformation | head shape, breed size)	EHR	5:1		1:2
Dental hygiene	E		Q, EHR		1:10	1.5:1
GA exam	U		EHR			1:10
Gingivitis	U		EHR			1:10
Head shape	D	EHR P (head shape | breed)	Q, EHR	1:10	1:2	1:10
Owner awareness	E					
Periodontitis	E	EHR P (periodontitis DX under COE or periodontitis DX under GA | age, breed, dental conformation, gingivitis, biofilm)		5:1		
Periodontitis DX			EHR			
Periodontitis DX under COE	E	EHR P (periodontitis DX under COE | conscious exam)	EHR	5:1		
Periodontitis DX under GA	E	EHR P (periodontitis DX under GA| GA exam)	EHR	5:1		
Pet compliance	E					

Where priors and dataset are defined as electronic health records (EHR), questionnaire (Q), expert input (E), demographic data (D), mapping (M) and uniform (U). Weights are ordered left to right as featured in the table. Other notation used: diagnosis (DX), conscious oral exam (COE), general anesthetic (GA), probability distribution (P).

The data for two nodes required special handling to satisfy the data quality criteria: (1) biofilm: experts provided the inverse conditional probability distribution to resample and correct biased dental hygiene data; (2) bad breath: questionnaire-derived probabilities were used to resample underreported EHR data.

Expert priors were elicited for nodes without sufficient observational data: bleeding gums, clinical signs, conscious exam, dental hygiene, owner awareness, periodontitis, both periodontitis diagnosis nodes (via COE and GA exam), and pet compliance. Where categories were simplified for elicitation, probabilities were interpolated across the full state space. Uniform priors were used for GA exam and gingivitis nodes to counteract small sample sizes resulting from high cardinality, created by multiple high cardinality parent variables. Priors for age, breed, breed size and head shape were integrated from the ‘demographic’ dataset from literature (43) to improve generalizability. Marginal data priors were calculated for nodes where only partial data was available (some parents were unobserved), but the data quality was sufficient. These were clinical signs, conscious exam, dental conformation, periodontitis diagnosis under COE, periodontitis diagnosis under GA.

A data prior was calculated for the periodontitis node, using the observed conditional probability of periodontitis diagnosis. This encoded observed relationships that could not be captured by expert opinion due to high cardinality of the CPT. Breed size and head shape were deterministic given breed in the data due to the data processing. Therefore, a data prior was added to the breed size node to encode uncertainty given breed. To mitigate sparseness in the posterior CPT, marginal data priors were added for nodes which had breed and at least one deterministically mapped node as parents. These were age, dental conformation, and head shape. Finally, a data prior for dental conformation was determined for specific breeds which had very small sample sizes in the data. This was calculated using the observed conditional probability of similar (same head shape and breed size) breeds.

### 3.4 Model evaluation

Individual node performance was evaluated by testing how well parent nodes predicted each child node’s state. This analysis included nine nodes with complete data for local DAGs: age, breed size, biofilm, bad breath, gingivitis, dental conformation, head shape, dental hygiene, and GA exam. Performance metrics showed considerable variation across nodes. Balanced accuracy averaged 52.0%, ranging from 6.8% (age, questionnaire dataset) to 93.8% (head shape, EHR dataset). ROC AUC averaged 74.4%, with head shape achieving 99.7% (EHR dataset) and dental hygiene only 46.8% (questionnaire dataset). The posterior probability certainty index (PPCI) averaged 0.51, ranging from 0.16 (bad breath, EHR dataset) to 0.93 (head shape, EHR dataset). For ordinal nodes (age, breed size, dental conformation, and dental hygiene), predictions were on average 0.3 categories away from true values. Full individual node predictive performance can be found in Supplementary Table 6.

Sensitivity analysis evaluated changes in the outcome variable before and after providing the evidence variable (entropy reduction). Gingivitis showed the strongest influence on periodontitis prediction (0.271), followed by clinical signs (0.146) and age (0.108). Six nodes showed minimal impact (<0.005): bad breath, GA exam, periodontitis diagnosis under COE, pet compliance, head shape and dental hygiene. Lower entropy reduction does not diminish the clinical importance of these nodes; as their effect may be captured elsewhere in the model. Supplementary Figure 1 presents the complete ranking of entropy reduction scores.

Expectation analysis evaluated how the model performed against 33 predefined hypotheses using both probabilistic and causal reasoning (Supplementary Table 7). Probabilistic reasoning correctly identified 32 of 33 expected outcomes, failing only to detect that giant breeds would have lower periodontitis risk than large breeds (Large: *p* = 0.064, Giant: *p* = 0.079, JSD = 0.019). Causal reasoning correctly identified 31 of 33 outcomes, missing that toy dogs would have higher periodontitis likelihood than small dogs (Toy: *p* = 0.163, Small: *p* = 0.166; JSD = 0.002), and that dolichocephalic dogs would have higher probability versus mesocephalic dogs (D: *p* = 0.121, M: *p* = 0.122, JSD = 0.001).

Predictive performance was assessed using balanced accuracy, ROC AUC, specificity, and sensitivity (Table 5). Periodontitis presence was predicted using a threshold probability of 0.151, calculated as the mean optimal threshold across all validation datasets. Prediction certainty varied across datasets; outcome uncertainty was measured using the PPCI with normalized thresholds. The observational EHR dataset achieved high certainty (PPCI = 0.856), the observational questionnaire was neutral (PPCI = 0.538), the expert survey and prospective study demonstrated high uncertainty 0.343 and 0.244, respectively. Complete PPCI values are presented in Table 5 and confusion matrices for all datasets available in Supplementary Figure 2.

**Table 5:** Overall predictive performance for the prediction of periodontitis performed across four datasets against an averaged optimal threshold of 0.151, measuring balanced accuracy, area under the receiver operating characteristic curve (ROC AUC), posterior probability certainty index (PPCI), sensitivity and specificity.

Dataset name	Average PPCI	Balanced accuracy	ROC AUC	Sensitivity	Specificity	Available model inputs
EHR	0.856	0.902	0.962	0.897	0.906	Age, Bad Breath, Biofilm, Breed, Breed Size, Clinical Signs, Conscious Exam, Dental Conformation, Dental Hygiene, GA Exam, Gingivitis, Head Shape
Questionnaire	0.538	0.687	0.790	0.639	0.735	Age, Bad Breath, Bleeding Gums, Breed, Breed Size, Clinical Signs, Dental Hygiene, Head Shape
Prospective study	0.244	0.606	0.583	0.913	0.300	Age, Bad Breath, Bleeding Gums, Breed, Clinical Signs, Conscious Exam, Dental Conformation, Dental Hygiene, GA Exam, Gingivitis
Expert survey	0.343	0.748	0.820	0.704	0.793	Age, Breed, Breed Size, Clinical Signs, Dental Hygiene, Head Shape

As true periodontitis is not available in data, metrics were calculated against a periodontitis DX from either conscious oral exam (COE) or under general anesthetic (GA). Where electronic health records (EHR).

### 3.5 Model results

The following analyses demonstrate practical model applications by examining how periodontitis probability varies with different inputs using both probabilistic and causal inference. All estimates targeted the periodontitis node rather than periodontitis diagnosis (relationship shown in Supplementary Figure 3). Table 6 summarizes probabilistic and causal estimates for each parent node individually, with effect sizes from pairwise comparisons between node states.

**Table 6:** Probability of periodontitis by varying exposure nodes using probabilistic and causal inference, with effect size estimated using Jensen-Shannon Divergence (JSD), in each case doing a pairwise comparison to the previous state, with the exception of breed, where all breeds are compared to mixed breed.

Node	Target	State	Probabilistic estimate	JSD	JSD effect size	Causal estimate	Causal JSD	Causal JSD effect size
No Evidence	Periodontitis	No evidence	0.124					
Age	Periodontitis	0–6 m	0.008			0.008		
	Periodontitis	3y	0.115	0.172	Strong	0.116	0.174	Strong
	Periodontitis	7y	0.277	0.147	Strong	0.271	0.140	Strong
	Periodontitis	11y	0.419	0.106	Strong	0.405	0.100	Moderate
	Periodontitis	13+	0.477	0.041	Weak	0.445	0.029	Weak
Bleeding gums	Periodontitis	False	0.094			0.124		
	Periodontitis	True	0.497	0.323	Very Strong	0.124	0	No effect
Breed	Periodontitis	Mixed Breed	0.115			0.115		
	Periodontitis	American Staffordshire Terrier	0.040	0.100	Strong	0.040	0.100	Strong
	Periodontitis	Chihuahua	0.217	0.098	Moderate	0.217	0.098	Moderate
	Periodontitis	German Shepherd	0.039	0.103	Strong	0.039	0.103	Strong
	Periodontitis	Labrador Retriever	0.052	0.081	Moderate	0.052	0.081	Moderate
	Periodontitis	Maltese	0.246	0.122	Strong	0.246	0.122	Strong
	Periodontitis	Poodle	0.156	0.043	Weak	0.156	0.043	Weak
	Periodontitis	Shih Tzu	0.201	0.084	Moderate	0.201	0.084	Moderate
	Periodontitis	Yorkshire Terrier	0.250	0.125	Strong	0.250	0.125	Strong
	Periodontitis	French Bulldog	0.126	0.013	Weak	0.126	0.013	Weak
	Periodontitis	Greyhound	0.293	0.159	Strong	0.293	0.159	Strong
	Periodontitis	Dachshund	0.252	0.127	Strong	0.252	0.127	Strong
	Periodontitis	American Cocker Spaniel	0.250	0.125	Strong	0.250	0.125	Strong
	Periodontitis	Rottweiler	0.045	0.092	Moderate	0.045	0.092	Moderate
	Periodontitis	Siberian Husky	0.047	0.089	Moderate	0.047	0.089	Moderate
	Periodontitis	Other	0.107	0.009	Weak	0.107	0.009	Weak
Breed size	Periodontitis	Toy	0.201			0.163		
	Periodontitis	Small	0.174	0.025	Weak	0.165	0.002	Weak
	Periodontitis	Medium small	0.138	0.035	Weak	0.153	0.012	Weak
	Periodontitis	Medium large	0.081	0.065	Moderate	0.138	0.015	Weak
	Periodontitis	Large	0.064	0.024	Weak	0.135	0.003	Weak
	Periodontitis	Giant	0.078	0.019	Weak	0.132	0.003	Weak
Clinical signs	Periodontitis	Good	0.053			0.124		
	Periodontitis	Medium	0.100	0.064	Moderate	0.124	0	No effect
	Periodontitis	Bad	0.637	0.410	Very Strong	0.124	0	No effect
Dental conformation	Periodontitis	Good	0.110			0.115		
	Periodontitis	Medium	0.182	0.072	Moderate	0.162	0.048	Weak
	Periodontitis	Bad	0.240	0.051	Moderate	0.185	0.021	Weak
Dental hygiene	Periodontitis	Good	0.136			0.121		
	Periodontitis	Medium	0.151	0.015	Weak	0.123	0.003	Weak
	Periodontitis	Bad	0.115	0.038	Weak	0.126	0.003	Weak
Gingivitis	Periodontitis	False	0.001			0.001		
	Periodontitis	True	0.470	0.443	Very Strong	0.298	0.337	Very Strong
Head shape	Periodontitis	Brachycephalic	0.148			0.131		
	Periodontitis	Mesocephalic	0.116	0.034	Weak	0.122	0.010	Weak
	Periodontitis	Dolichocephalic	0.133	0.018	Weak	0.121	0.001	Weak
Biofilm	Periodontitis	False	0.004			0.019		
	Periodontitis	True	0.176	0.240	Very Strong	0.137	0.163	Strong

The probability of periodontitis given no evidence is 0.124.

Probability of periodontitis was assessed for the most common breeds and those identified as high risk in literature. As breed had no parent nodes both probabilistic and causal inference gave the same estimates. The highest risk breeds were Greyhound (*p* = 0.293), Dachshund (*p* = 0.252) and Yorkshire Terrier (*p* = 0.250). The lowest risk breeds were American Staffordshire Terrier (*p* = 0.040), Labrador Retriever (*p* = 0.052) and Rottweiler (*p* = 0.045). Breed probabilities were compared to Mixed Breed (*p* = 0.115) to estimate effect size: JSDs varied between 0.039 (German Shepherd vs. Mixed Breed) and 0.159 (Greyhound vs. Mixed Breed), with an average of 0.155.

Using probabilistic inference, periodontitis probability increased with age: 0–6 months *p* = 0.008, rising at 3 years to *p* = 0.115, at 7 years *p* = 0.277, at 11 years *p* = 0.419, and at 13 years *p* = 0.477. JSD values for consecutive age comparisons based on probabilistic inference were 0.172, 0.147, and 0.106 (all strong), with only the 11–13-year comparison showing weak divergence (0.041). Causal inference reported the same trend, similar absolute probabilities and JSDs.

Breed size effects generally showed higher periodontitis probability in smaller dogs for both inference types. However, exceptions emerged: probabilistic inference showed giant breeds with higher probability than large breeds (0.078 vs. 0.064), whilst causal inference showed small dogs with higher probability than toy dogs (0.165 vs. 0.163). All pairwise comparisons by increasing size yielded weak JSDs (0.002–0.035) for both inference types, except medium-small versus medium-large in probabilistic inference (0.065, moderate).

Head shape effects were minimal for both inference types. Probabilistic inference showed risk increasing from mesocephalic (lowest) through dolichocephalic to brachycephalic (highest), whilst causal inference showed a different pattern mesocephalic and dolichocephalic approximately equal and brachycephalic highest. For probabilistic inference, comparing brachycephalic to mesocephalic increased risk 1.28-fold (JSD 0.034, weak), for causal this was 1.07-fold (JSD 0.010, weak). Comparing mesocephalic to dolichocephalic also resulted in weak effect sizes for both probabilistic and causal inference (JSDs 0.018 and 0.001, respectively).

**Figure 3:**
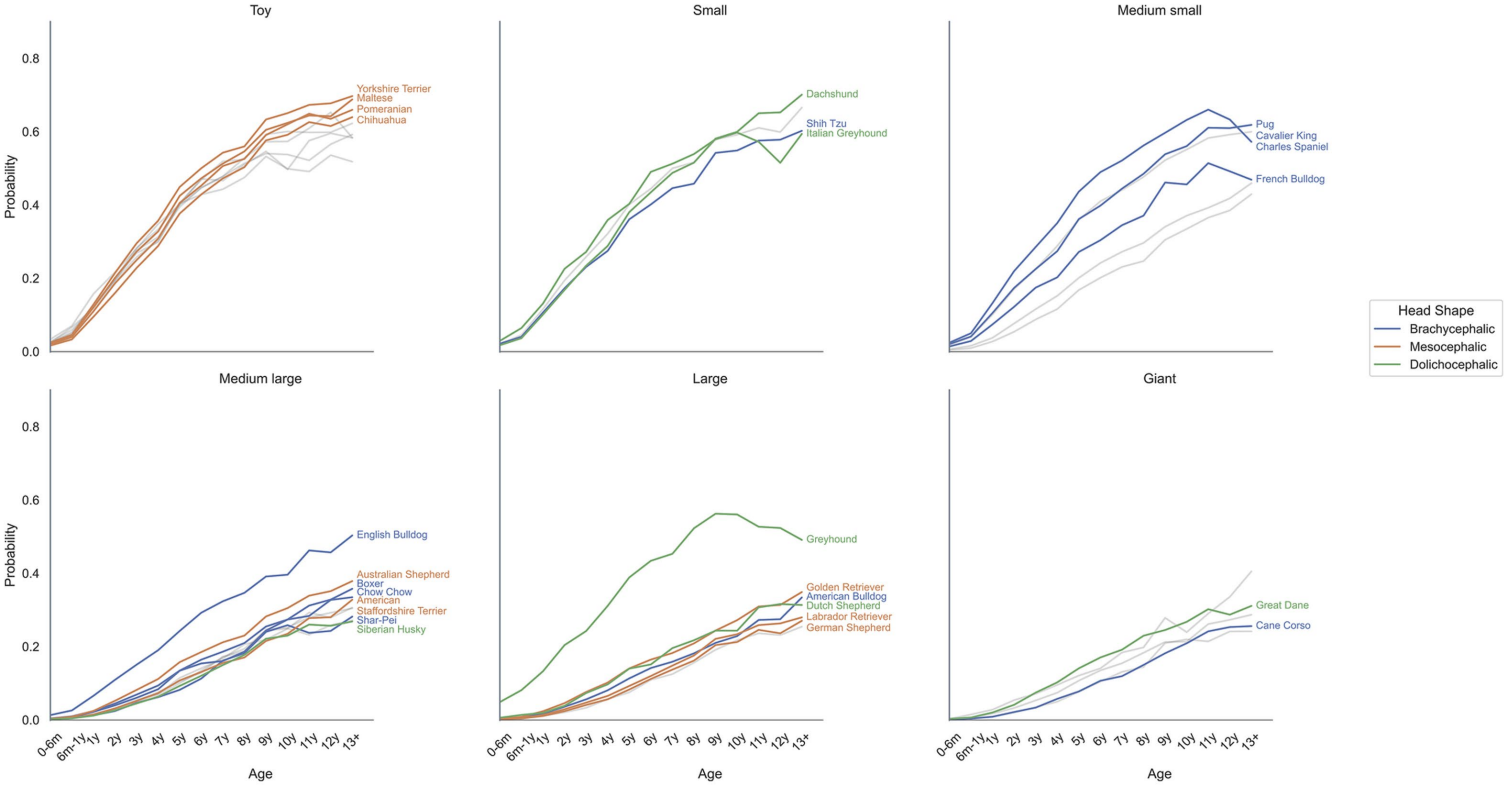
Probability of periodontitis by age across different breeds. Probabilistic inference was used to estimate periodontitis probability for each breed. Within each breed size and head shape category, up to 10 most prevalent breeds are labeled and colored by head shape. Breeds outside the top 10 are shown in grey.

The model was queried for the 10 most common breeds within each head shape category, using breed and age as inputs to generate probability curves for periodontitis across the given age ranges (Figure 3). These probabilistic calculations showed periodontitis likelihood increasing with age across all breeds, with smaller breeds generally showing higher probabilities than larger breeds within each age group.

Dental conformation increased periodontitis probability from good (probabilistic *p* = 0.11, causal *p* = 0.115) to bad (probabilistic *p* = 0.240, causal *p* = 0.185). The pairwise comparisons in increasing rank resulted in moderate effect sizes for probabilistic inference (JSDs 0.072 and 0.051, respectively) and weak effect sizes for causal inference (JSDs 0.048 and 0.021, respectively). For probabilistic inference, comparing bad (overcrowding, rotation) dental conformation to good resulted in a fold-change of 2.18.

**Figure 4:**
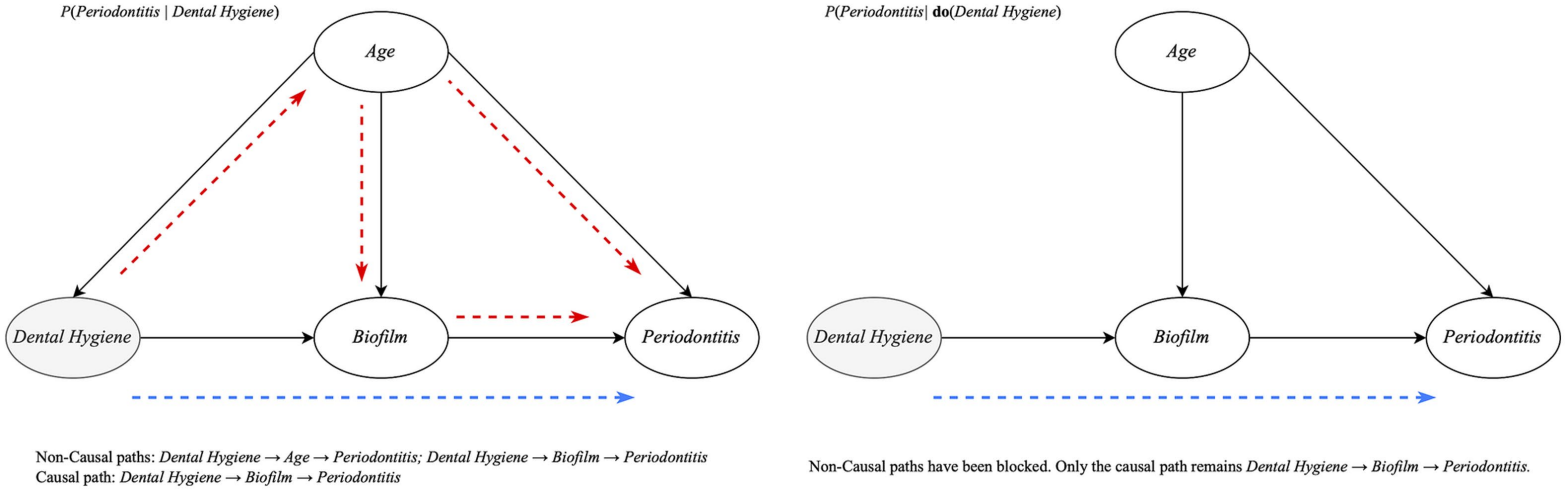
Directed acyclic subgraph showing causal (blue dashed line) and non-causal paths (red dashed line) between the level of dental hygiene and periodontitis. Showing probabilistic inference on the left subgraph and causal inference on the right.

Dental hygiene provided weak effects between groups and conflicting responses depending on inference type. Probabilistic inference resulted in estimates of *p* = 0.136 for good, *p* = 0.151 for medium and *p* = 0.115 for bad. Causal inference estimated increasing probability of periodontitis with decreasing dental hygiene: *p* = 0.121 for good, *p* = 0.123 for medium and *p* = 0.126 for bad. Figure 4 shows the subgraph and applied inference between the two approaches.

Presence of biofilm increased periodontitis probability whilst absence decreased it for both probabilistic and causal inference. Comparing presence (*p* = 0.176) and absence (*p* = 0.004) for probabilistic inferences there was a 44-fold change difference (JSD = 0.24, very strong), for causal inference (*p* = 0.137 versus *p* = 0.019) there was a 7.21-fold change difference (JSD = 0.163, strong).

Evidence of gingivitis increased periodontitis probability whilst absence decreased it, for both probabilistic and causal inference. Comparing presence (*p* = 0.470) and absence (*p* = 0.001) for probabilistic inferences there was a 470-fold change difference (JSD = 0.443, very strong), for causal inference (*p* = 0.298 versus *p* = 0.001) there was a 298-fold change difference (JSD = 0.337, very strong).

As clinical signs and bleeding gums are both children of the periodontitis node, only probabilistic inferences could be made. For clinical signs, periodontitis probability increased from good (*p* = 0.053) through medium (*p* = 0.100) to bad (*p* = 0.637), with pairwise comparisons of increasing rank indicating moderate (good vs. medium, JSD 0.064) to very strong (medium vs. bad, JSD 0.41) effect sizes. For bleeding gums, presence increased periodontitis probability to 0.497, whilst absence decreased it to 0.094, comparing presence and absence resulted in a 5.29-fold difference (JSD = 0.323, very strong).

The previous examples examined input variables individually; however, a realistic application requires inference with multiple simultaneous inputs. Table 7 includes examples of how periodontitis probability updates with incremental evidence for an example breed (Shih Tzu), comparing probabilistic and causal inference approaches.

**Table 7:** Probability of periodontitis by varying exposure of parent nodes using probabilistic and causal inference, with effect size estimated using Jensen-Shannon Divergence (JSD), in each case doing a pairwise comparison to the base scenario.

Scenario	Breed	Age	Dental conformation	Biofilm	Gingivitis	Probabilistic estimate	JSD	JSD effect size	Causal estimate	Causal JSD	Causal JSD effect size
No evidence	-	-	-	-	-	0.124					
Base scenario	Shih Tzu	-	-	-	-	0.201			0.201		
1	Shih Tzu	4y	-	-	-	0.275	0.062	Moderate	0.275	0.062	Moderate
2	Shih Tzu	12y	-	-	-	0.578	0.278	Very Strong	0.578	0.278	Very Strong
3	Shih Tzu	12y	Bad	-	-	0.605	0.297	Very Strong	0.605	0.297	Very Strong
4	Shih Tzu	12y	-	True	-	0.591	0.287	Very Strong	0.591	0.287	Very Strong
5	Shih Tzu	12y	-	-	True	0.921	0.545	Very Strong	0.915	0.539	Very Strong
6	Shih Tzu	12y	Bad	True	True	0.946	0.572	Very Strong	0.946	0.572	Very Strong

The probability of periodontitis given no evidence is 0.124. Where evidence for a node was not provided it is indicated with "-".

## 4 Discussion

This study represents the culmination of a substantial multi-method hybrid research project, integrating large observational datasets, prospective study data, and expert elicitation. The outputs are a novel DAG articulating causal relationships between risk factors for periodontal disease in dogs, and a Bayesian network parameterised to function as both a probabilistic and causal risk model.

The DAG revealed periodontal disease as a complex multifactorial condition with numerous risk factors contributing to cascading causal relationships. Base risk begins with breed, a non-modifiable factor determining both breed size and skull shape. These morphological features influence dental conformation, how teeth align and fit within the mouth. Bad dental conformation promotes biofilm accumulation, which can be reduced through preventive measures like tooth brushing or professional care. Without effective intervention and as pets age, plaque accumulates, becomes increasingly complex, produces odor, and increases the likelihood of immune responses leading to gingivitis. If untreated, gingivitis can progress to periodontitis. While both conditions can be associated with clinical signs such as bleeding gums, oral pain, odor, and behavioral changes, gingival inflammation is the cause of bleeding. Whether these signs result in diagnosis depends on two pathways: owner awareness leading to veterinary visits, and the type of examination performed (conscious or under anesthesia). The structure encoded within the DAG captures our assumptions about how biological, behavioral, and other factors relating to care interact to determine disease probability.

This causal structure was parameterised into a Bayesian network containing over 33,200 conditional probabilities, using EHR data from over nine million dogs, demographic data, owner surveys, and expert consultation. Any of the resulting 19 nodes can serve as inputs or outputs for both probabilistic and causal inference. Unlike other analytical models, Bayesian networks provide value through their structure as well as predictions, evaluating solely on single outcomes underestimates the network’s full analytical potential. The hybrid nature of this model also presented unique validation challenges: no single dataset contained all required variables, precluding traditional holdout validation, and validating against individual data sources would be counterproductive since the model was specifically designed to overcome their inherent biases. Therefore, multiple validation strategies were implemented.

First, conditional independence testing validated the DAG structure. Whilst node development followed an iterative, subjective process, this objective structural validation tested whether the proposed relationships existed within the data. This confirmed the DAG structure and identified two previously overlooked interactions: between age and dental hygiene, and between age and clinical signs. These were incorporated into the final model.

Second, the trained model was validated using unseen data from the observational datasets to assess whether it had learned meaningful patterns. Performance varied by node (biofilm outperformed age) and dataset (EHR data outperformed questionnaires). Perfect accuracy was not the target, as predicting parent nodes from child nodes is inherently limited. The model also aimed to learn general patterns rather than overfit to specific datasets. For example, incorporating external demographic priors for age increased generalisability but likely reduced accuracy when predicting for the EHR data.

Model sensitivity was examined through entropy reduction analysis, measuring how much uncertainty decreased when specific evidence was provided. The model showed greatest sensitivity to gingivitis, clinical signs, and age. Unsurprising given the proximity of the nodes to periodontitis within the network, and the expected underlying mechanism whereby previous episodes of gingivitis lead to increased risk of periodontitis, as well as clinical signs providing evidence of existing disease. Beyond statistical validation, the model’s outputs were also verified against epidemiological hypotheses using predefined scenarios, with the majority producing expected results (Supplementary Table 7).

Finally, the model’s predictive accuracy as a periodontitis classifier was tested using four independent datasets, each containing different variable subsets. Perfect accuracy was not expected given inherent limitations: available variables fell short of clinical examination data, and datasets contained known biases including underdiagnosis in EHRs, self-reporting errors in questionnaires, and sampling bias introduced from the data source. The model performed consistently well across three datasets. Sensitivity against the questionnaire dataset was relatively low. However, the validation was performed by classifying periodontitis versus the broader category of reported periodontal disease within this dataset. As such, the model was unlikely to correctly classify some of the positive cases. The prospective study showed poorest performance, incorrectly classifying seven of ten healthy pets as having periodontitis. However, these misclassifications involved cases with known gingivitis, missing breed data, or high-risk breeds, suggesting the model appropriately weighted risk factors despite incorrect overall classification. Future work could explore optimization for such edge cases.

Having validated that the model performed as expected, it could be used as both a probabilistic and causal inference tool for any node within the network. This capability was demonstrated by examining how different evidence and inference types affected periodontitis probability. The two inference types address fundamentally different questions: probabilistic inference calculates probabilities based on the evidence, capturing associations. Causal inference, using Pearl’s ‘do’ operator, intervenes in the graph to block confounding pathways, isolating direct causal effects. Essentially, probabilistic inference describes ‘what is observed’ whilst causal inference reveals ‘what would happen if we intervened’. This distinction is crucial for clinical decision-making, as probabilistic estimates reflect the patterns observed in practice, whilst causal estimates indicate the expected impact of interventions.

The model correctly differentiated between high and low-risk breeds, with differences shown between breed, breed size, and age (Figure 3). These probabilities fall within expected ranges and ratios reported in other studies (2, 17), although those studies targeted periodontal disease whilst this study focused on periodontitis specifically. Age showed the expected strong relationship with periodontitis. Both probabilistic and causal inference showed probability increasing from near zero in puppies to 10% in youth and approximately 47% by age 13, also mirroring trends and ratios reported in independent studies (2, 17).

The model captured expected relationships between morphological features and disease risk (1, 2), with smaller dogs and brachycephalic breeds showing increased risk. However, effect sizes for both breed size and head shape were modest and occasionally inconsistent, varying by inference type. These inconsistencies reflect complex confounding within the DAG structure and fundamental differences between inference approaches. Probabilistic inference captures statistical associations between breed size and periodontitis. In contrast, causal inference isolates breed size effects independent of breed, creating implausible scenarios (toy-sized Rottweilers or giant Chihuahuas) that reveal why these approaches diverge. In probabilistic inference, breed size affects periodontitis indirectly through breed, age, head shape, and dental conformation; these indirect effects are modest but contain complex relationships making them not independent of each other. For example, small breed dogs are predominantly brachycephalic (60) whilst smaller long-nosed dogs tend to live longer (61). Thus, breed size correlates with both head shape and longevity, which each influence periodontitis risk through different mechanisms. Breed prevalence creates additional confounding: Greyhounds dominate the dolichocephalic group (49) with high periodontitis rates, but this also reflects their advanced average age. Conversely, French Bulldogs show unexpectedly low rates despite being brachycephalic, as their population skews young due to recent popularity (62) and relatively short lifespans (63). These examples demonstrate why distinguishing association from causation is crucial. Causal inference blocks indirect pathways to isolate specific effects, revealing ‘what if’ scenarios that sometimes contradict observed associations. This distinction, illustrated here through implausible breed modifications, becomes essential when evaluating real interventions and attempting to predicting their impact.

Dental conformation, a child of age, breed size, and head shape, was defined to capture the general morphological health of teeth. Base risk derives from skull size and shape (64, 65), then increases with exposure time through age. Whilst the parent nodes demonstrated complexity in probability interpretation, dental conformation behaved as expected, with risk doubling from good to bad conformation. Similarly, biofilm presence also increased periodontitis probability, confirming the established relationship between biofilm and periodontitis development (7).

The presence of gingivitis showed the strongest effect, increasing periodontitis probability over 300-fold from base levels to near zero when absent to high probability when present. Whilst this magnitude of effect has not been previously reported, prospective longitudinal studies have documented associations between gingivitis and periodontitis (19, 20). The temporal precedence and substantial impact of conformation, biofilm, and gingivitis on probability estimates highlight their potential as early biomarkers for periodontitis risk. These clinical signs are readily observable during routine examinations, and potentially by pet owners with guidance (14), making them valuable surrogates for identifying at-risk patients before irreversible tissue damage occurs. Furthermore, whilst observed differences between high and low-risk categories diminish under causal inference compared to probabilistic, considerable risk reduction persists when shifting from ‘observed’ to ‘what if’. This confirms what is known in practice that these factors are not only biomarkers but also viable intervention targets.

A final demonstration of the network’s utility was its ability to integrate multiple evidence variables simultaneously. This was illustrated using a Shih Tzu breed use case, where periodontitis probability shifted with accumulating observations. With breed alone, baseline probability was moderate (*p* = 0.201). Adding age updated the risk profile: a young Shih Tzu (4 years) showed low probability (*p* = 0.275), whilst an older Shih Tzu (12 years) showed higher probability (*p* = 0.57). Further incorporating clinical evidence of bad dental conformation (*p* = 0.684), biofilm presence (*p* = 0.660), and gingivitis (*p* = 0.924) showed each factor increased risk independently, with combined evidence raising probability to *p* = 0.962. This demonstrates how the network synthesizes complex, multivariate clinical presentations for individualized risk assessment. These predictions might help contextualize the potential value of different preventive strategies for individual patients. Also of note is the minimal differences observed between probabilistic and causal inference within these scenarios a result of the evidence covering all parent nodes of periodontitis.

A critical component of network development involved precise node definitions. For instance, the biofilm node was conceptually defined as ‘Any kind of visible biofilm on teeth, e.g., plaque or calculus (tartar)’. However, translating this concept required dataset-specific interpretations to ensure interoperability across sources with different recording conventions. Whilst this definition worked for expert elicitation, it differs from typical electronic health record entries. Equally important was articulating causal relationships between nodes, making assumptions explicit and testable. Our threshold for causality required plausible biological mechanisms, supported by evidence where available or consensus opinion where not. Given this inherent subjectivity, documenting both the reasoning process and underlying assumptions was essential—a practice we recommend for others pursuing similar approaches.

Incorporating dental hygiene presented a particular challenge. Data on preventive routines were sparse in the observational datasets, necessitating substantial expert input to populate this node. The concept of preventative care itself is inherently complex, as both timing and efficacy of interventions significantly impact outcomes (24, 66, 67). Using a three-level ranked node to represent all dental hygiene activities likely oversimplified the nuanced relationships between intervention type, frequency, and effectiveness. Additionally, the temporal dynamics required further resolution: whilst periodontal disease was defined as ‘ever diagnosed’, dental hygiene required temporal precedence that proved difficult to establish. Although meticulous plaque removal can help prevent periodontal disease even in genetically predisposed patients, it cannot improve attachment loss in cases of already established periodontitis so the temporal relationship between instituting home care and development of periodontitis is critical. This temporal ambiguity, combined with our simplified node structure, may explain why dental hygiene showed neutral impact on network outcomes. These limitations highlight important methodological considerations for future work incorporating preventative interventions.

Expert elicitation presented logistical challenges. Identifying independent veterinary experts proved difficult, as specialists in periodontal disease are relatively few and their expertise in high demand. Ideally, we would have fully sampled our network and variable space, including formal assessment of inter and intra-rater variability. However, practical constraints necessitated strategic decisions about elicitation scope: we ensured at least two experts per table and conducted individual rather than group interviews to accommodate complex schedules, geographic constraints, and varying timelines. Expert responses were averaged, with participants given opportunity to review individual and consensus outputs and provide feedback. Whilst alternative elicitation approaches exist with potential advantages (68, 69), our approach balanced feasibility constraints with methodological rigor.

By demonstrating how hybrid Bayesian networks can augment large-scale observational data with clinical expertise to overcome individual source limitations, this work intends to contribute to advancing quantitative risk assessment in veterinary epidemiology. It provides a prototype for addressing other complex, multifactorial diseases where data quality, availability, or diagnostic challenges have historically impeded comprehensive understanding. The tool functions as both a predictive model for real-world pets understanding their absolute and relative risk dependent on observations but also as an investigative tool to understand intervention outcomes.

## Data Availability

The original contributions presented in the study are included in the article/Supplementary material, further inquiries can be directed to the corresponding author.
