# Supplementary material for: Risk assessment for canine periodontal disease using a hybrid causal Bayesian network
Source: Front Vet Sci. 2026 Apr 23;13:1781228. doi: 10.3389/fvets.2026.1781228 (PMC13149179; doi:10.3389/fvets.2026.1781228)

Supplementary Material

**Supplementary Table 1: Detail of hypotheses supporting the validated directed acyclic graph (Figure 1) constructed to capture knowledge and assumptions related to periodontitis. Each edge is described between parent and child nodes. Where diagnosis (DX), conscious oral exam (COE) and general anaesthetic (GA).**

| **Node** | **Description** |
| --- | --- |
| **Dental conformation:** Ranked node for the position of the teeth within a dog’s mouth, created by an additive rating of attributes related to dental conformation. | |
| Periodontitis | It is hypothesized that overcrowding, occlusion, or reduction in gingiva and alveolar bone ​^1,2^​ may contribute to risk of periodontitis. Increased risk may also arise from increased gaps in-between teeth, exposed gums, or lack of saliva motility ^3,4^. |
| Biofilm | Poor dental conformation, such as overcrowding or malocclusion, may prevent effective chewing and cleaning, or limit saliva motility, leading to build-up of biofilm ^5^. |
| **Head shape:**  The morphology of the dog’s skull: brachycephalic, mesocephalic or dolichocephalic. | |
| GA exam | Brachycephalic dogs may be less likely to undergo GA due to increased risk of complications ^6,7^. |
| Dental conformation | Brachycephalic breeds are more likely to have overcrowding, malocclusion, reduction in gingiva and alveolar bone^8^. Dolichocephalic breeds are more likely to have increased gaps between teeth, exposed gums and lack of saliva motility. |
| **Dental hygiene:** Ranked node for the level of dental hygiene activities, created by an additive rating of attributes related to dental hygiene. | |
| Biofilm | Dental hygiene activities should limit the build-up of biofilm. |
| **Breed:** The assigned breed of a pet. | |
| Dental conformation | Certain breeds exhibit features that lead to poor dental conformation, independent of head shape alone. |
| Head shape | Head shape is determined by breed; in general, each breed has a defined head shape. |
| Breed size | Breed size is a property of breed. |
| Conscious oral exam | It is hypothesized that type and likelihood of exam may depend on breed. Conscious oral exams may be deprioritized for breeds that have alternative health issues^9^ or may be prioritized for those unlikely to undergo GA for examination. |
| Owner awareness | Consulted veterinary experts advised that an owner’s awareness of dental health may vary dependent on breed; veterinarians and breeders may be more likely to educate owners of breeds with expected predisposition for oral health issues. |
| Age | Life expectancy is dependent on breed^10,11^. |
| Periodontitis | Breed influences the host’s response to infection, representing differences in genetic or immune affects not from head shape or via plaque ^12^. |
| Gingivitis | Breed influences the host’s response to infection, representing differences in genetic or immune affects not from head shape or via plaque ^12^. |
| Pet compliance | The genetics of breeds causes differences in their behaviour influencing pet compliance during visits to a veterinary hospital^13^. |
| **Pet compliance:** Whether a dog complies with veterinary examination. | |
| Conscious oral exam | A dog’s compliance affects the likelihood that a conscious oral exam will be performed. |
| **Breed size:** The average size for a breed, categorized into toy, small, medium small, medium large, large, giant. Accounts for shared attributes at a size level above breed. | |
| Head shape | Head shape varies by breed size, such that likelihood to have a specific head shape is dependent on breed size. |
| Age | Life expectancy varies by breed size ^14,15^. |
| Dental conformation | Brachycephalic morphology is more frequent in smaller sized dogs compared to large sized dogs leading to poor dental conformation. Small breeds have proportionally larger teeth than larger breeds and therefore predisposed to overcrowding ^16^. |
| **Biofilm**: Any kind of visible biofilm on teeth, e.g., plaque or calculus (tartar). | |
| Bad breath | The build-up of biofilm, indicative of increased bacterial load, is a cause of bad breath. |
| Gingivitis | The biofilm forming in plaque is the stimulus that triggers the host immune system response which then develops into gingivitis^17,18^. |
| Periodontitis | The biofilm forming in plaque is the stimulus that triggers the host immune system response which triggers periodontitis ^17,18^. |
| **Bad breath:** An offensive odor coming from the mouth, most commonly caused by volatile sulfur compounds produced by bacteria | |
| Owner awareness | Bad breath may call attention to a dog’s oral health, thereby increasing the owner’s awareness of dental issues. |
| **Owner awareness:** Owner is alerted to their pet having the potential to having periodontitis; owner understands their pets associated risk of periodontitis. | |
| COE | It is hypothesized that an owner with awareness of oral health would be more likely to proactively seek a veterinary visit for an oral exam. |
| **COE:** Whether the dog’s oral cavity has been examined by a veterinarian whilst conscious, within the year prior to evaluation. | |
| GA exam | A conscious oral exam often precedes a GA exam, in identifying the need for follow-up. |
| Periodontitis diagnosis under a COE | A diagnosis of periodontitis may be made at a conscious oral exam, based on visual indications. |
| **GA exam:** A comprehensive oral exam under general anaesthetic including radiography and/or probing, within the year prior to evaluation. | |
| Periodontitis diagnosis under GA | A definitive diagnosis of periodontitis often requires examination under GA, allowing greater opportunity for assessment. |
| **Age:** The age of the dog at assessment. | |
| Dental hygiene | Likelihood to participate in dental hygiene activities may depend on the age of the dog. Professional treatment may not be recommended for younger dogs, and compliance to at-home brushing may vary with age. |
| Biofilm | Once deciduous teeth have foliated, the build-up of biofilm happens over time; as such, biofilm is more likely to be present in older dogs. |
| Conscious oral exam | The likelihood for a conscious oral examination to occur may depend on age; younger dogs may be more likely to have regular check-ups, and older pets at risk of GA complications may be more likely to have a conscious exam. |
| Periodontitis | Increased age may result in accumulation of exposure or damage^19^. It is also linked to immune system deteriorating with age^20^. |
| Periodontitis diagnosis | Opportunity for diagnosis increases with age if the exposure to the veterinarian over time increases; older dogs may be treated with higher suspicion of oral health issues by veterinarians. |
| Gingivitis | Increased age may result in accumulation of exposure or damage. It is also linked to immune system deteriorating with age. |
| Clinical signs | Increased age may result in accumulation of damage, increasing the likelihood of displaying clinical signs. |
| **Periodontitis diagnosis under a COE:** Whether the dog ever had a record of diagnosis of any stages (1-4) of periodontitis by a veterinarian under conscious oral examination. | |
| GA exam | An initial diagnosis made via conscious oral examination may be followed up by an examination under GA to fully assess the severity of the disease. |
| **Periodontitis diagnosis under GA:** The diagnosis of periodontitis as a result of an examination under GA, likely to be more accurately detected and assessed. | |
| **Periodontitis:** Whether the dog has any periodontal disease stage 2-4, regardless of diagnosis. | |
| Periodontitis diagnosis under COE | The presence of disease may lead to a diagnosis during a conscious oral examination. |
| Periodontitis diagnosis under GA | The presence of disease may lead to a diagnosis during an examination under GA. |
| Clinical signs | Presence of the disease increases the occurrence of clinical signs. |
| Bleeding gums | The presence of periodontitis causes bleeding gums. |
| **Gingivitis:** The dog has received a gingivitis diagnosis by a veterinarian. | |
| Bleeding gums | The presence of gingivitis causes bleeding gums^21^. |
| Periodontitis | Gingivitis, unmanaged, may lead to periodontitis^22^. |
| **Bleeding gums:** Owner has observed the dog has bleeding gums when eating or chewing or has noticed blood on toys. | |
| Owner awareness | The presence of bleeding gums is a clinical sign that an owner is likely to notice and infer oral issues. |
| Clinical signs | Bleeding gums is often the first signs of gingivitis, which left untreated will precede the clinical signs of periodontitis. |
| **Clinical signs:** Ranked node for severity of clinical signs of periodontitis, as observed by an owner or professional. | |
| Owner awareness | The presence of clinical signs may increase the owner’s awareness of oral health issues. |

1. Hale, F. Stop brachycephalism, now! *The Canadian Veterinary Journal* **54**, 185 (2013).

2. Hale, F. A. Dental and Oral Health for the Brachycephalic Companion Animal. *Health and Welfare of Brachycephalic (Flat-faced) Companion Animals* 235–250 (2021) doi:10.1201/9780429263231-14.

3. Kumar, A. *et al.* ISP good clinical practice recommendations for gum care. *J Indian Soc Periodontol* **27**, 4 (2023).

4. Vallabhan, C. G. *et al.* Assessment of salivary flow rate in patients with chronic periodontitis. *J Pharm Bioallied Sci* **12**, S308–S312 (2020).

5. Bernhardt, O. *et al.* New insights in the link between malocclusion and periodontal disease. *J Clin Periodontol* **46**, 144–159 (2019).

6. Salt, C., Morrison, J. A., Spofford, N. & O’Rourke, A. Data-driven safety limits for assessing perianesthetic mortality risk in dogs and cats undergoing elective procedures. *Vet Anaesth Analg* https://doi.org/10.1016/J.VAA.2025.03.008 (2025) doi:10.1016/J.VAA.2025.03.008.

7. Gruenheid, M. *et al.* Risk of anesthesia-related complications in brachycephalic dogs. *J Am Vet Med Assoc* **253**, 301–306 (2018).

8. Geiger, M. *et al.* Exceptional Changes in Skeletal Anatomy under Domestication: The Case of Brachycephaly. *Integrative Organismal Biology* **3**, obab023 (2021).

9. O’Neill, D. G. *et al.* Epidemiology of periodontal disease in dogs in the UK primary‐care veterinary setting. *J Small Anim Pract* **62**, 1051 (2021).

10. Montoya, M. *et al.* Life expectancy tables for dogs and cats derived from clinical data. *Front Vet Sci* **10**, 1082102 (2023).

11. Teng, K. T. yun, Brodbelt, D. C., Pegram, C., Church, D. B. & O’Neill, D. G. Life tables of annual life expectancy and mortality for companion dogs in the United Kingdom. *Sci Rep* **12**, 1–11 (2022).

12. Harvey, C. E. Management of Periodontal Disease: Understanding the Options. *Veterinary Clinics of North America: Small Animal Practice* **35**, 819–836 (2005).

13. Duffy, D. L., Hsu, Y. & Serpell, J. A. Breed differences in canine aggression. *Appl Anim Behav Sci* **114**, 441–460 (2008).

14. Greer, K. A., Canterberry, S. C. & Murphy, K. E. Statistical analysis regarding the effects of height and weight on life span of the domestic dog. *Res Vet Sci* **82**, 208–214 (2007).

15. Patronek, G. J., Waters, D. J. & Glickman, L. T. Comparative longevity of pet dogs and humans: implications for gerontology research. *J Gerontol A Biol Sci Med Sci* **52**, (1997).

16. Pegram, C. *et al.* Frequency, breed predisposition and demographic risk factors for overweight status in dogs in the UK. *Journal of Small Animal Practice* **62**, 521–530 (2021).

17. Kinane, D. F., Stathopoulou, P. G. & Papapanou, P. N. Periodontal diseases. *Nat Rev Dis Primers* **3**, (2017).

18. Abdulkareem, A. A. *et al.* Current concepts in the pathogenesis of periodontitis: from symbiosis to dysbiosis. *J Oral Microbiol* **15**, 2197779 (2023).

19. Wallis, C. & Holcombe, L. J. A review of the frequency and impact of periodontal disease in dogs. *Journal of Small Animal Practice •* **61**, (2020).

20. Blount, D. G., Pritchard, D. I. & Heaton, P. R. Age-related alterations to immune parameters in Labrador retriever dogs. *Vet Immunol Immunopathol* **108**, 399–407 (2005).

21. Niemiec, B. *Veterinary Periodontology*. (John Wiley & Sons, 2012).

22. Harvey, C. E. Periodontal disease in dogs. Etiopathogenesis, prevalence, and significance. *Vet Clin North Am Small Anim Pract* **28**, (1998).

**Supplementary Table 2: Definitions within the electronic health records (EHR) and questionnaire data for each node in the directed acyclic graph (Figure 1), describing risk factors associated with periodontitis. Node definitions based on EHR data were constructed in collaboration with veterinary experts.**

| **Node** | **EHR definition** | | **Questionnaire definition** | **Time preceding outcome** |
| --- | --- | --- | --- | --- |
| Periodontitis | Not available | Not available | |  |
| Periodontitis diagnosis | Diagnosis of any of “Periodontal Disease”, “Periodontal Disease Stage 2”, “Periodontal Disease Stage 3”, “Periodontal Disease Stage 4” or “Periodontal Pocket” | Selection of “Periodontal disease” in the dental diagnosis section of the oral health questionnaire. Required. | | N/A (outcome) |
| Gingivitis diagnosis | Diagnosis of “Gingivitis” or “Periodontal Disease Stage 1” or a symptom record of “Infected Pockets in Gums” | Not available | | Any time |
| Bleeding gums | Not available | Selection of “Bloody saliva” in the dental observations section of the oral health questionnaire. Required. | | Any time |
| Bad breath | Symptom record of “Bad Breath” | Selection of “Bad breath” in the dental observations section or selection of “Mildly offensive” or “Very offensive” in the breath description question of the oral health questionnaire. Required | | Within one-year |
| Biofilm | Diagnosis of “Dental Calculus” or “Dental Calculus, Subgingival”, or symptom record of “Tartar on Teeth” or “Tartar Right” | Not available | | Within one-year |
| Conscious oral exam | Visit reason of “Dental/Oral”, “Dental Recheck”, "Comprehensive Exam", "Examination/Consultation", "New Puppy/Kitten Exam", "Examination",  "Adopted Pet Exam", "New Friend Exam", "Castration w/ Comprehensive Exam", "Ovariohysterectomy w/ Comprehensive Exam" | Not available | | Within one-year |
| Exam under general anaesthetic (GA) | Visit reason of “Dental Cleaning w/ Comprehensive Exam”, “Dental Cleaning w/ Oral Surgery” or “Dental Cleaning”.  Checked that the GA went ahead from billing information and details of surgery cancellation in medical notes. | Not available | | Within one-year |
| Abnormal eruption | Diagnosis of “Tooth, Impacted” | Not available | | Any time |
| Overcrowding | Diagnosis of “Tooth, Supernumerary” [sic] or symptom record of “Supernumerary Teeth” or “Supernumerary Teeth Right” | Not available | | Any time |
| Rotation | Diagnosis of “Tooth, Rotated” | Not available | | Any time |
| Retained deciduous teeth | Diagnosis of “Deciduous Teeth, Retained” | Data available but not sufficient | | Any time |
| Malocclusion | Diagnosis of “Crossbite Anterior”, “Malocclusion”, “Malocclusion, Class 1”, “Malocclusion, Class 2”, “Malocclusion, Class 3”, “Overbite”, “Underbite” or “Wry Bite”, or symptom record of “Maligned Teeth” | Data available but not sufficient | | Any time |
| Tooth loss | Item billing record of type “Dental Surgery” and description matching “extract”, “remov” or “tooth loss”. | Not available | | Any time |
| Tooth mobility | Diagnosis of “Incisors, Loose”, “Premolar, Loose”, “Molars, Loose” or “Canine loose” | Not available | | Within one-year |
| Gum recession | Diagnosis of “Gingival Recession” or symptom record of “Receded Gums” | Not available | | Any time |
| Lethargy | Diagnosis of “Lethargy” | Not available | | Within one-year |
| Discomfort eating | Symptom record of “Trouble Swallowing” | Selection of “Soft chewable treats” in the treat preference section of the microbiome questionnaire or selection of “Pain when eating as exhibited by dropping food or chewing on one side” in the dental observations section of the oral health questionnaire. Not required. | | Within one-year |
| Head shy | Not available | Selection of “Resistance to being touched on the head” in the dental observations section oral health questionnaire. Required. | | Within one-year |
| Professional treatment (dental) | Visit reason of “Dental Cleaning w/ Comprehensive Exam”, “Dental Cleaning” or “Dental Cleaning w/ Oral Surgery”, or item billing record of type “Dental Prophylaxis” or item description of “Root Planing” or “Teeth Brushing” | Response of “1-2 times” or “>3 times” to the question of “How often have you had [pet] treated (including preventative) for dental problems” in the oral health questionnaire. Required. | | Any time |
| Tooth brushing | Symptom record of “Teeth Cleaning Frequency” or item billing record of “Toothbrush” or “Toothpaste” | Selection of “Toothbrush & toothpaste” in the dental health products section of the oral health questionnaire. Required. | | Any time |
| Dental chews | Item billing record in category “Dental” matching “chew”, “greenies” or “dentee” | Selection of “Dental treats or chews” in the treat preference section of the microbiome questionnaire or response of “Multiple times each day”, “Daily”, “Weekly” or “Monthly” in the dental chew treats frequency section of the oral health questionnaire. Required. | | Any time |
| Diet | Item billing record of type “Dental” and subtype “Diet” | Selection of “Pill or tablet”, “Liquid, gel or oil”, “Powder” or “Other” in the dental health products section of the oral health questionnaire. Required. | | Any time |
| Dental toys | Not available | Selection of “Chew toys” or “Long lasting chews” in the dental health products section of the oral health questionnaire. Required. | | Any time |

Supplementary Table 3: Directed acyclic graph sub nodes scored according to its value of contribution to the rank node, defined in collaboration with a veterinarian expert in canine oral health. E.g. rotation scores 3 indicating, if present, a strong negative impact on its overall dental conformation. Thresholds determine, when sub node scores are combined, which rank value a pet would take.

| **Sub node** | **Score** |
| --- | --- |
| **Dental conformation (≥ 0 = good, ≥ 1 = medium, ≥ 3 = bad)** | |
| Abnormal eruption | 1 |
| Overcrowding | 3 |
| Rotation | 3 |
| Retained deciduous teeth | 1 |
| Malocclusion | 1 |
| **Clinical signs (≥ 0 = good, ≥ 1 = medium, ≥ 4 = bad)** | |
| Tooth loss | 100 |
| Tooth mobility | 100 |
| Gum recession | 100 |
| Lethargy | 1 |
| Discomfort eating | 2 |
| Head shy | 1 |
| **Dental hygiene (≥ 0 = bad, ≥ 1 = medium, ≥ 3 = good)** | |
| Professional treatment | 100 |
| Tooth brushing | 100 |
| Dental chews | 2 |
| Diet | 1 |
| Dental toys | 1 |

Supplementary Table 4: Head shape to breed size mapping, all other breeds were assigned “Null”.

| **Brachycephalic** | **Mesocephalic** | **Dolichocephalic** |
| --- | --- | --- |
| American Bulldog, Boston Terrier, Boxer, Brussels Griffon, Bullmastiff, Cane Corso, Cavalier King Charles Spaniel, Chow Chow, English Bulldog, French Bulldog, Japanese Chin, Lhasa Apso, Pekingese, Pug, Shar-Pei, Shih Tzu, Tibetan Spaniel | Airedale Terrier, Akita, Alaskan Malamute, American Cocker Spaniel, American Eskimo, American Staffordshire Terrier, Artesian Normand Basset, Australian Cattle Dog, Australian Shepherd, Australian Terrier, Basenji, Basset Hound, Beagle, Bearded Collie, Belgian Malinois, Belgian Sheepdog, Belgian Tervuren, Bernese Mountain Dog, Bichon Frise, Bloodhound, Border Collie, Border Terrier, Bouvier Des Flandres, Boykin Spaniel, Brittany Spaniel, Cairn Terrier, Catahula Leopard Dog, Chesapeake Bay Retriever, Chihuahua, Chinese Crested, Cock-A-Poo, Coton de Tulear, Dalmatian, Doberman Pinscher, English Cocker Spaniel, English Pointer, English Setter, English Shepherd, English Springer Spaniel, Flat Coat Retriever, Fox Terrier, German Pointer, German Shepherd, Giant Poodle, Giant Schnauzer, Golden Retriever, Gordon Setter, Great Pyrenees, Havanese, Hound, Hungarian Vizsla, Irish Setter, Jack Russell Terrier, Keeshond, Labrador Retriever, Lancashire Heeler, Lhasa-Poo, Maltese, Manchester Terrier, Mastiff, Medium Poodle, Miniature Pinscher, Miniature Poodle, Miniature Schnauzer, Newfoundland, Norwegian Elkhound, Old English Sheepdog, Papillon, Pomeranian, Poodle, Portuguese Water Dog, Rat Terrier, Rhodesian Ridgeback, Rottweiler, Saint Bernard, Samoyed, Schipperke, Scottish Terrier, Shetland Sheepdog, Shiba Inu, Silky Terrier, Soft-Coated Wheaten Terrier, Staffordshire Bull Terrier, Standard Poodle, Standard Schnauzer, Teacup Poodle, Terrier, Tibetan Terrier, Toy Fox Terrier, Toy Poodle, Weimaraner, Welsh Corgi, Welsh Terrier, West Highland White Terrier, Yorkshire Terrier. | Afghan Hound, Bull Terrier, Collie, Dachshund, Dutch Shepherd, Great Dane, Greyhound, Italian Greyhound, Miniature Dachshund, Rabbit Dachshund, Siberian Husky, Whippet |

**Supplementary Table 5: Model breed input categorized into approximate low, expected and high risk of that breed having periodontitis determined by our expert elicitation panel**

| **Low** | **Typical** | **High** |
| --- | --- | --- |
| Australian Cattle Dog  Belgian Malinois  Border Collie  Cane Corso  Dalmatian  German Shepherd  Labrador Retriever  Rottweiler  Shar-Pei  Siberian Husky | American Bulldog  American Staffordshire Terrier  Australian Shepherd  Beagle  Boxer  Bullmastiff  Chow Chow  Cock-A-Poo  Dutch Shepherd  Golden Retriever  Great Dane  Mastiff  Mixed Breed  Other  Poodle | American Cocker Spaniel  Bichon Frise  Cavalier King Charles Spaniel  Chihuahua  Chinese Crested  Coton de Tulear  Dachshund  English Bulldog  French Bulldog  Greyhound  Havanese  Italian  Greyhound  Maltese  Papillon  Pomeranian  Pug  Shih Tzu  Silky Terrier  Yorkshire Terrier |

***Supplementary Table 6: Individual node predictive performance for a node given itself and its parents were all available in the given observational dataset. The metrics, for binary nodes these were balanced accuracy, and* area under the receiver operating characteristic curve *(ROC AUC), for multiclass nodes these were balanced accuracy and one-vs-one ROC AUC, for ordinal nodes number (N) of classed from True. If complete parent-child datasets were available average posterior probability certainty index (PPCI) was calculated. The periodontitis diagnosis node has been excluded from evaluation as this node is a direct map of its parents. Where electronic health records (EHR)***

| **Metric name** | **Node Name** | **Metric Value** | **Dataset Name** | **Parents** |
| --- | --- | --- | --- | --- |
| Balanced Accuracy | Age | 0.072 | EHR | ['Breed', 'Breed Size'] |
| Average PPCI | Age | 0.686 | EHR | ['Breed', 'Breed Size'] |
| N Classes from True Average | Age | 0.450 | EHR | ['Breed', 'Breed Size'] |
| N Classes from True 0-6M | Age | 0.003 | EHR | ['Breed', 'Breed Size'] |
| N Classes from True 6M-1Y | Age | 0.068 | EHR | ['Breed', 'Breed Size'] |
| N Classes from True 1Y | Age | 0.125 | EHR | ['Breed', 'Breed Size'] |
| N Classes from True 2Y | Age | 0.180 | EHR | ['Breed', 'Breed Size'] |
| N Classes from True 3Y | Age | 0.248 | EHR | ['Breed', 'Breed Size'] |
| N Classes from True 4Y | Age | 0.314 | EHR | ['Breed', 'Breed Size'] |
| N Classes from True 5Y | Age | 0.381 | EHR | ['Breed', 'Breed Size'] |
| N Classes from True 6Y | Age | 0.448 | EHR | ['Breed', 'Breed Size'] |
| N Classes from True 7Y | Age | 0.514 | EHR | ['Breed', 'Breed Size'] |
| N Classes from True 8Y | Age | 0.580 | EHR | ['Breed', 'Breed Size'] |
| N Classes from True 9Y | Age | 0.648 | EHR | ['Breed', 'Breed Size'] |
| N Classes from True 10Y | Age | 0.713 | EHR | ['Breed', 'Breed Size'] |
| N Classes from True 11Y | Age | 0.780 | EHR | ['Breed', 'Breed Size'] |
| N Classes from True 12Y | Age | 0.844 | EHR | ['Breed', 'Breed Size'] |
| N Classes from True 13+ | Age | 0.911 | EHR | ['Breed', 'Breed Size'] |
| ROC AUC | Age | 0.550 | EHR | ['Breed', 'Breed Size'] |
| Balanced Accuracy | Age | 0.068 | Questionnaire | ['Breed', 'Breed Size'] |
| Average PPCI | Age | 0.680 | Questionnaire | ['Breed', 'Breed Size'] |
| N Classes from True Average | Age | 0.458 | Questionnaire | ['Breed', 'Breed Size'] |
| N Classes from True 0-6M | Age | 0.000 | Questionnaire | ['Breed', 'Breed Size'] |
| N Classes from True 6M-1Y | Age | 0.067 | Questionnaire | ['Breed', 'Breed Size'] |
| N Classes from True 1Y | Age | 0.129 | Questionnaire | ['Breed', 'Breed Size'] |
| N Classes from True 2Y | Age | 0.187 | Questionnaire | ['Breed', 'Breed Size'] |
| N Classes from True 3Y | Age | 0.260 | Questionnaire | ['Breed', 'Breed Size'] |
| N Classes from True 4Y | Age | 0.321 | Questionnaire | ['Breed', 'Breed Size'] |
| N Classes from True 5Y | Age | 0.400 | Questionnaire | ['Breed', 'Breed Size'] |
| N Classes from True 6Y | Age | 0.456 | Questionnaire | ['Breed', 'Breed Size'] |
| N Classes from True 7Y | Age | 0.520 | Questionnaire | ['Breed', 'Breed Size'] |
| N Classes from True 8Y | Age | 0.590 | Questionnaire | ['Breed', 'Breed Size'] |
| N Classes from True 9Y | Age | 0.660 | Questionnaire | ['Breed', 'Breed Size'] |
| N Classes from True 10Y | Age | 0.709 | Questionnaire | ['Breed', 'Breed Size'] |
| N Classes from True 11Y | Age | 0.794 | Questionnaire | ['Breed', 'Breed Size'] |
| N Classes from True 12Y | Age | 0.854 | Questionnaire | ['Breed', 'Breed Size'] |
| N Classes from True 13+ | Age | 0.920 | Questionnaire | ['Breed', 'Breed Size'] |
| ROC AUC | Age | 0.534 | Questionnaire | ['Breed', 'Breed Size'] |
| Balanced Accuracy | Breed Size | 0.808 | EHR | ['Breed'] |
| Average PPCI | Breed Size | 0.743 | EHR | ['Breed'] |
| N Classes from True Average | Breed Size | 0.060 | EHR | ['Breed'] |
| N Classes from True Toy | Breed Size | 0.040 | EHR | ['Breed'] |
| N Classes from True Small | Breed Size | 0.064 | EHR | ['Breed'] |
| N Classes from True Medium Small | Breed Size | 0.021 | EHR | ['Breed'] |
| N Classes from True Medium Large | Breed Size | 0.036 | EHR | ['Breed'] |
| N Classes from True Large | Breed Size | 0.035 | EHR | ['Breed'] |
| N Classes from True Giant | Breed Size | 0.166 | EHR | ['Breed'] |
| ROC AUC | Breed Size | 0.968 | EHR | ['Breed'] |
| Balanced Accuracy | Breed Size | 0.804 | Questionnaire | ['Breed'] |
| Average PPCI | Breed Size | 0.722 | Questionnaire | ['Breed'] |
| N Classes from True Average | Breed Size | 0.058 | Questionnaire | ['Breed'] |
| N Classes from True Toy | Breed Size | 0.017 | Questionnaire | ['Breed'] |
| N Classes from True Small | Breed Size | 0.063 | Questionnaire | ['Breed'] |
| N Classes from True Medium Small | Breed Size | 0.000 | Questionnaire | ['Breed'] |
| N Classes from True Medium Large | Breed Size | 0.072 | Questionnaire | ['Breed'] |
| N Classes from True Large | Breed Size | 0.027 | Questionnaire | ['Breed'] |
| N Classes from True Giant | Breed Size | 0.167 | Questionnaire | ['Breed'] |
| ROC AUC | Breed Size | 0.964 | Questionnaire | ['Breed'] |
| Balanced Accuracy | Biofilm | 0.806 | EHR | ['Age', 'Dental Conformation', 'Dental Hygiene'] |
| Average PPCI | Biofilm | 0.391 | EHR | ['Age', 'Dental Conformation', 'Dental Hygiene'] |
| ROC AUC | Biofilm | 0.865 | EHR | ['Age', 'Dental Conformation', 'Dental Hygiene'] |
| Balanced Accuracy | Bad Breath | 0.500 | EHR | ['Biofilm'] |
| Average PPCI | Bad Breath | 0.156 | EHR | ['Biofilm'] |
| ROC AUC | Bad Breath | 0.641 | EHR | ['Biofilm'] |
| Balanced Accuracy | Gingivitis | 0.627 | EHR | ['Age', 'Breed', 'Biofilm'] |
| Average PPCI | Gingivitis | 0.445 | EHR | ['Age', 'Breed', 'Biofilm'] |
| ROC AUC | Gingivitis | 0.833 | EHR | ['Age', 'Breed', 'Biofilm'] |
| Balanced Accuracy | Dental Conformation | 0.333 | EHR | ['Breed', 'Breed Size', 'Head Shape'] |
| Average PPCI | Dental Conformation | 0.596 | EHR | ['Breed', 'Breed Size', 'Head Shape'] |
| N Classes from True Average | Dental Conformation | 0.333 | EHR | ['Breed', 'Breed Size', 'Head Shape'] |
| N Classes from True Bad | Dental Conformation | 0.667 | EHR | ['Breed', 'Breed Size', 'Head Shape'] |
| N Classes from True Medium | Dental Conformation | 0.333 | EHR | ['Breed', 'Breed Size', 'Head Shape'] |
| N Classes from True Good | Dental Conformation | 0.000 | EHR | ['Breed', 'Breed Size', 'Head Shape'] |
| ROC AUC | Dental Conformation | 0.746 | EHR | ['Breed', 'Breed Size', 'Head Shape'] |
| Balanced Accuracy | Head Shape | 0.938 | EHR | ['Breed', 'Breed Size'] |
| Average PPCI | Head Shape | 0.927 | EHR | ['Breed', 'Breed Size'] |
| ROC AUC | Head Shape | 0.997 | EHR | ['Breed', 'Breed Size'] |
| Balanced Accuracy | Head Shape | 0.878 | Questionnaire | ['Breed', 'Breed Size'] |
| Average PPCI | Head Shape | 0.908 | Questionnaire | ['Breed', 'Breed Size'] |
| ROC AUC | Head Shape | 0.994 | Questionnaire | ['Breed', 'Breed Size'] |
| Balanced Accuracy | Dental Hygiene | 0.333 | EHR | ['Age'] |
| Average PPCI | Dental Hygiene | 0.291 | EHR | ['Age'] |
| N Classes from True Average | Dental Hygiene | 0.333 | EHR | ['Age'] |
| N Classes from True Bad | Dental Hygiene | 0.000 | EHR | ['Age'] |
| N Classes from True Medium | Dental Hygiene | 0.333 | EHR | ['Age'] |
| N Classes from True Good | Dental Hygiene | 0.667 | EHR | ['Age'] |
| ROC AUC | Dental Hygiene | 0.600 | EHR | ['Age'] |
| Balanced Accuracy | Dental Hygiene | 0.333 | Questionnaire | ['Age'] |
| Average PPCI | Dental Hygiene | 0.225 | Questionnaire | ['Age'] |
| N Classes from True Average | Dental Hygiene | 0.333 | Questionnaire | ['Age'] |
| N Classes from True Bad | Dental Hygiene | 0.000 | Questionnaire | ['Age'] |
| N Classes from True Medium | Dental Hygiene | 0.333 | Questionnaire | ['Age'] |
| N Classes from True Good | Dental Hygiene | 0.667 | Questionnaire | ['Age'] |
| ROC AUC | Dental Hygiene | 0.468 | Questionnaire | ['Age'] |
| Balanced Accuracy | Dental Hygiene | 0.333 | Expert Survey | ['Age'] |
| Average PPCI | Dental Hygiene | 0.218 | Expert Survey | ['Age'] |
| N Classes from True Average | Dental Hygiene | 0.333 | Expert Survey | ['Age'] |
| N Classes from True Bad | Dental Hygiene | 0.000 | Expert Survey | ['Age'] |
| N Classes from True Medium | Dental Hygiene | 0.333 | Expert Survey | ['Age'] |
| N Classes from True Good | Dental Hygiene | 0.667 | Expert Survey | ['Age'] |
| ROC AUC | Dental Hygiene | 0.501 | Expert Survey | ['Age'] |
| Balanced Accuracy | Dental Hygiene | 0.333 | Prospective Survey | ['Age'] |
| Average PPCI | Dental Hygiene | 0.190 | Prospective Survey | ['Age'] |
| N Classes from True Average | Dental Hygiene | 0.333 | Prospective Survey | ['Age'] |
| N Classes from True Bad | Dental Hygiene | 0.000 | Prospective Survey | ['Age'] |
| N Classes from True Medium | Dental Hygiene | 0.333 | Prospective Survey | ['Age'] |
| N Classes from True Good | Dental Hygiene | 0.667 | Prospective Survey | ['Age'] |
| ROC AUC | Dental Hygiene | 0.662 | Prospective Survey | ['Age'] |
| Balanced Accuracy | GA Exam | 0.631 | EHR | ['Age', 'Conscious Exam', 'Head Shape', 'Periodontitis DX Under COE'] |
| Average PPCI | GA Exam | 0.495 | EHR | ['Age', 'Conscious Exam', 'Head Shape', 'Periodontitis DX Under COE'] |
| ROC AUC | GA Exam | 0.841 | EHR | ['Age', 'Conscious Exam', 'Head Shape', 'Periodontitis DX Under COE'] |

**Supplementary Figure 1: Entropy reduction score for directed acyclic graph defined in Figure 1, measuring the uncertainty of each node when predicting periodontitis. Where conscious oral exam (COE), diagnosis (DX) and general anesthetic (GA).**


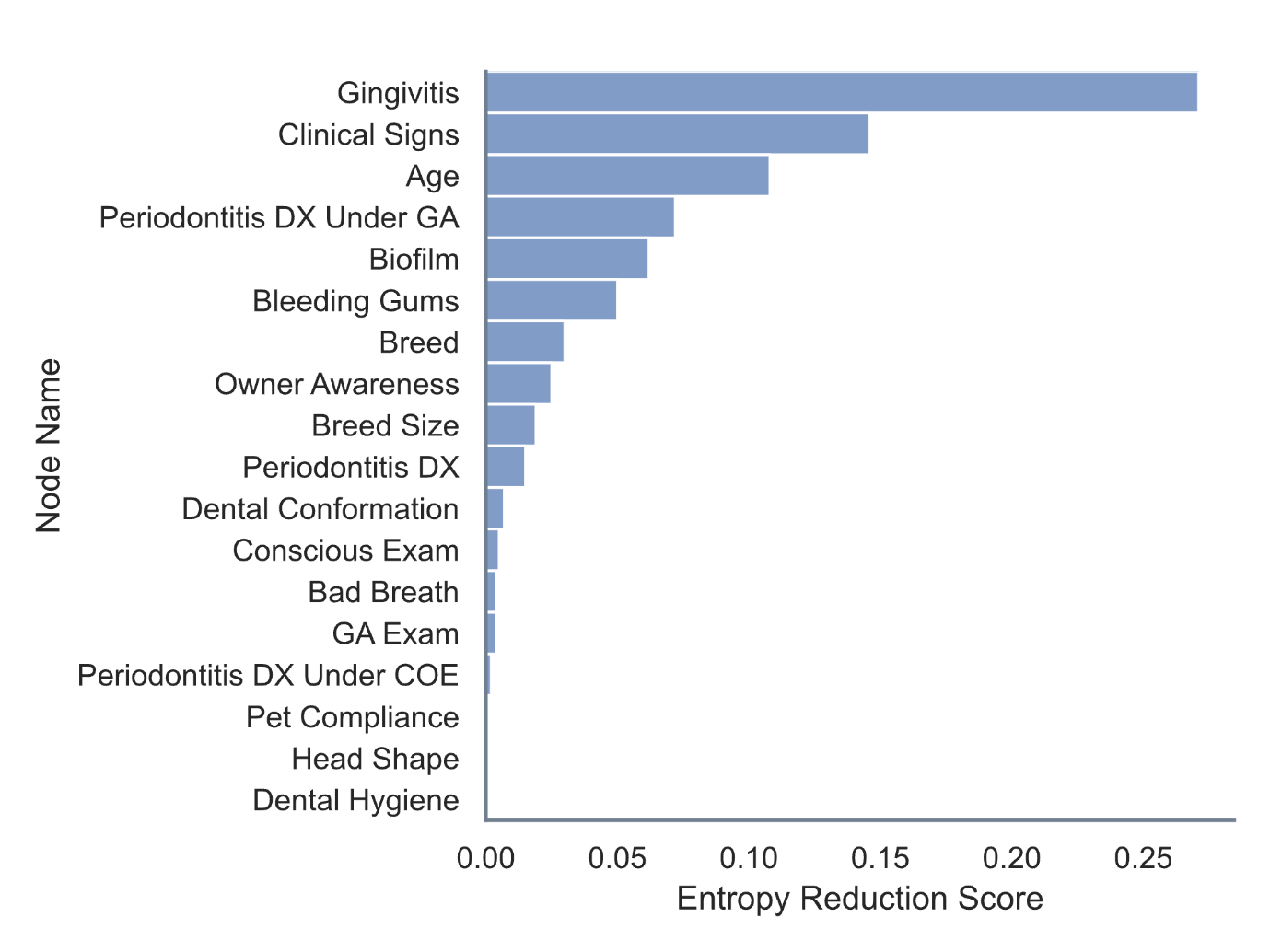


**Supplementary Table 7: Expectation analysis results, testing predefined hypotheses against model results, both probabilistic and causal inferences. The Jensen-Shannon Divergence (JSD) gives an estimate of the uncertainty and significance of the comparisons. The “result” column indicates if the comparison of the estimates agrees with the hypothesis.**

|  |  | **Probabilistic results** | | | | **Causal results** | | | |
| --- | --- | --- | --- | --- | --- | --- | --- | --- | --- |
| **Hypothesis** | **Sub-Hypothesis** | **Estimates** | **JSD** | **JSD Effect Size** | **Result** | **Estimates** | **JSD** | **JSD Effect Size** | **Result** |
| Older age increases the risk of periodontitis | 3y > 0-6m | 0-6m: 0.008, 3y: 0.115 | 0.172 | Strong | True | 0-6m: 0.008, 3y: 0.116 | 0.174 | Strong | True |
|  | 7y > 3y | 3y: 0.115, 7y: 0.277 | 0.147 | Strong | True | 3y: 0.116, 7y: 0.271 | 0.140 | Strong | True |
|  | 11y > 7y | 7y: 0.277, 11y: 0.42 | 0.106 | Strong | True | 7y: 0.271, 11y: 0.405 | 0.100 | Strong | True |
|  | 13+ > 11y | 11y 0.42, 13+: 0.472 | 0.041 | Weak | True | 11y: 0.405, 13+: 0.445 | 0.029 | Weak | True |
| Smaller breeds have a higher risk of periodontitis | Toy > Small | Toy 0.202, Small 0.174 | 0.025 | Weak | True | Toy: 0.163, Small: 0.166 | 0.002 | Weak | False |
|  | Small > Medium small | Small: 0.174, Medium small: 0.138 | 0.035 | Weak | True | Small: 0.166, Medium small: 0.153 | 0.012 | Weak | True |
|  | Medium small > Medium large | Medium small: 0.138, Medium large: 0.08 | 0.065 | Moderate | True | Medium small: 0.153, Medium large: 0.137 | 0.015 | Weak | True |
|  | Medium large > Large | Medium large: 0.08, Large: 0.064 | 0.024 | Weak | True | Medium large: 0.137, Large: 0.135 | 0.003 | Weak | True |
|  | Large > Giant | Large: 0.064, Giant: 0.079 | 0.019 | Weak | False | Large: 0.135, Giant: 0.132 | 0.003 | Weak | True |
| Worse dental conformation increases the risk of periodontitis | Bad > Medium | Bad: 0.24, Medium: 0.181 | 0.051 | Moderate | True | Bad: 0.185, Medium: 0.162 | 0.021 | Weak | True |
|  | Medium > Good | Medium: 0.181, Good: 0.11 | 0.072 | Moderate | True | Medium: 0.162, Good: 0.115 | 0.048 | Weak | True |
| Presence of plaque increases the probability of periodontitis | True > False | True: 0.177, False: 0.004 | 0.240 | Very Strong | True | True: 0.137, False: 0.019 | 0.163 | Strong | True |
| Head shape impacts the risk of periodontitis | Brachycephalic > Mesocephalic | B: 0.148, M; 0.116 | 0.034 | Weak | True | B: 0.131, M: 0.122 | 0.010 | Weak | True |
|  | Dolichocephalic > Mesocephalic | D: 0.133, M: 0.116 | 0.018 | Weak | True | D: 0.121, M: 0.122 | 0.001 | Weak | False |
| Worse dental hygiene increases the probability of periodontitis | Bad > Medium | Bad: 0.117, Medium: 0.114 | 0.003 | Weak | True | Bad: 0.118, Medium: 0.116 | 0.003 | Weak | True |
|  | Medium > Good | Medium: 0.114, Good: 0.111 | 0.003 | Weak | True | Medium: 0.116, Good: 0.113 | 0.003 | Weak | True |
| High risk breeds have a higher risk of periodontitis than low risk breeds | High risk (Greyhound) > low risk (Australian Cattle Dog) | Greyhound: 0.293, Australian Cattle Dog: 0.071 | 0.209 | Very Strong | True | Greyhound: 0.293, Australian Cattle Dog: 0.071 | 0.209 | Very Strong | True |
|  | High risk (Greyhound) > low risk (Siberian Husky) | Greyhound: 0.293, Siberian Husky: 0.047 | 0.242 | Very Strong | True | Greyhound: 0.293, Siberian Husky: 0.047 | 0.242 | Very Strong | True |
|  | High risk (Greyhound) > low risk (Labrador Retriever) | Greyhound: 0.293, Labrador Retriever: 0.052 | 0.235 | Very Strong | True | Greyhound: 0.293, Labrador Retriever: 0.052 | 0.235 | Very Strong | True |
|  | High risk (Greyhound) > low risk (Rottweiler) | Greyhound: 0.293, Rottweiler: 0.045 | 0.245 | Very Strong | True | Greyhound: 0.293, Rottweiler: 0.045 | 0.245 | Very Strong | True |
|  | High risk (Chihuahua) > low risk (Australian Cattle Dog) | Chihuahua: 0.217, Australian Cattle Dog: 0.071 | 0.150 | Strong | True | Chihuahua: 0.217, Australian Cattle Dog: 0.071 | 0.150 | Strong | True |
|  | High risk (Chihuahua) > low risk (Siberian Husky) | Chihuahua: 0.217, Siberian Husky: 0.047 | 0.184 | Strong | True | Chihuahua: 0.217, Siberian Husky: 0.047 | 0.184 | Strong | True |
|  | High risk (Chihuahua) > low risk (Labrador Retriever) | Chihuahua: 0.217, Labrador Retriever: 0.052 | 0.177 | Strong | True | Chihuahua: 0.217, Labrador Retriever: 0.052 | 0.177 | Strong | True |
|  | High risk (Chihuahua) > low risk (Rottweiler) | Chihuahua: 0.217, Rottweiler: 0.045 | 0.188 | Strong | True | Chihuahua: 0.217, Rottweiler: 0.045 | 0.188 | Strong | True |
|  | High risk (Yorkshire Terrier) > low risk (Australian Cattle Dog) | Yorkshire Terrier: 0.248, Australian Cattle Dog: 0.071 | 0.176 | Strong | True | Yorkshire Terrier: 0.248, Australian Cattle Dog: 0.071 | 0.176 | Strong | True |
|  | High risk (Yorkshire Terrier) > low risk (Siberian Husky) | Yorkshire Terrier: 0.25, Siberian Husky: 0.047 | 0.210 | Very Strong | True | Yorkshire Terrier: 0.248, Siberian Husky: 0.047 | 0.210 | Very Strong | True |
|  | High risk (Yorkshire Terrier) > low risk (Labrador Retriever) | Yorkshire Terrier: 0.25, Labrador Retriever: 0.052 | 0.203 | Very Strong | True | Yorkshire Terrier: 0.248, Labrador Retriever: 0.052 | 0.203 | Very Strong | True |
|  | High risk (Yorkshire Terrier) > low risk (Rottweiler) | Yorkshire Terrier: 0.25, Rottweiler: 0.045 | 0.13 | Very Strong | True | Yorkshire Terrier: 0.248, Rottweiler: 0.045 | 0.213 | Very Strong | True |
| Risk of periodontitis is higher when plaque is present, given gingivitis is present | True > False | True: 0.472, False: 0.259 | 0157 | Strong | True | True: 0.324, False: 0.183 | 0.115 | Strong | True |
| Worse dental conformation increases the probability of plaque | Bad > Medium | Bad: 0.846, Medium: 0.821 | 0.024 | Weak | True | Bad: 0.837, Medium: 0.816 | 0.020 | Weak | True |
|  | Medium > Good | Medium: 0.821, Good: 0.673 | 0.121 | Strong | True | Medium: 0.816, Good: 0.674 | 0.115 | Strong | True |
| Worse dental hygiene increases the probability of gingivitis | Bad > Medium | Bad: 0.316, Medium: 0.309 | 0.006 | Weak | True | Bad: 0.316, Medium: 0.308 | 0.006 | Weak | True |
|  | Medium > Good | Medium: 0.309, Good: 0.300 | 0.007 | Weak | True | Medium: 0.308, Good: 0.299 | 0.007 | Weak | True |

Supplementary Figure 2: Confusion matrices illustrating predictive performance of periodontitis across four evaluation datasets, electronic health records (EHR), questionnaire, expert survey and prospective study. The classification performance was determined on a threshold of 0.151, the optimal threshold across all datasets


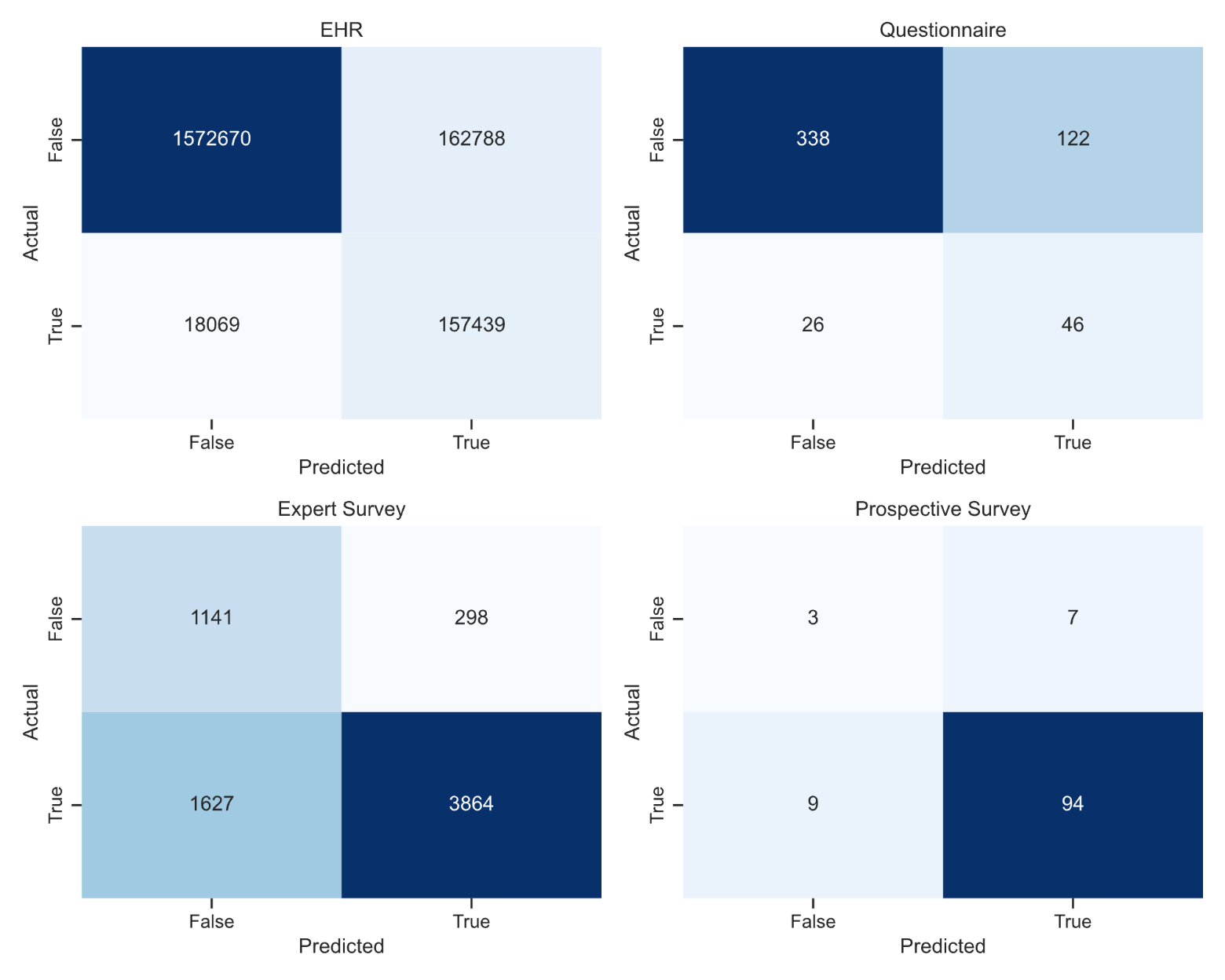


Supplementary Figure 3: Probabilistic probability of having periodontitis and having a diagnosis (DX) from a given method, under conscious oral exam (COE) or general anesthetic (GA) by age.


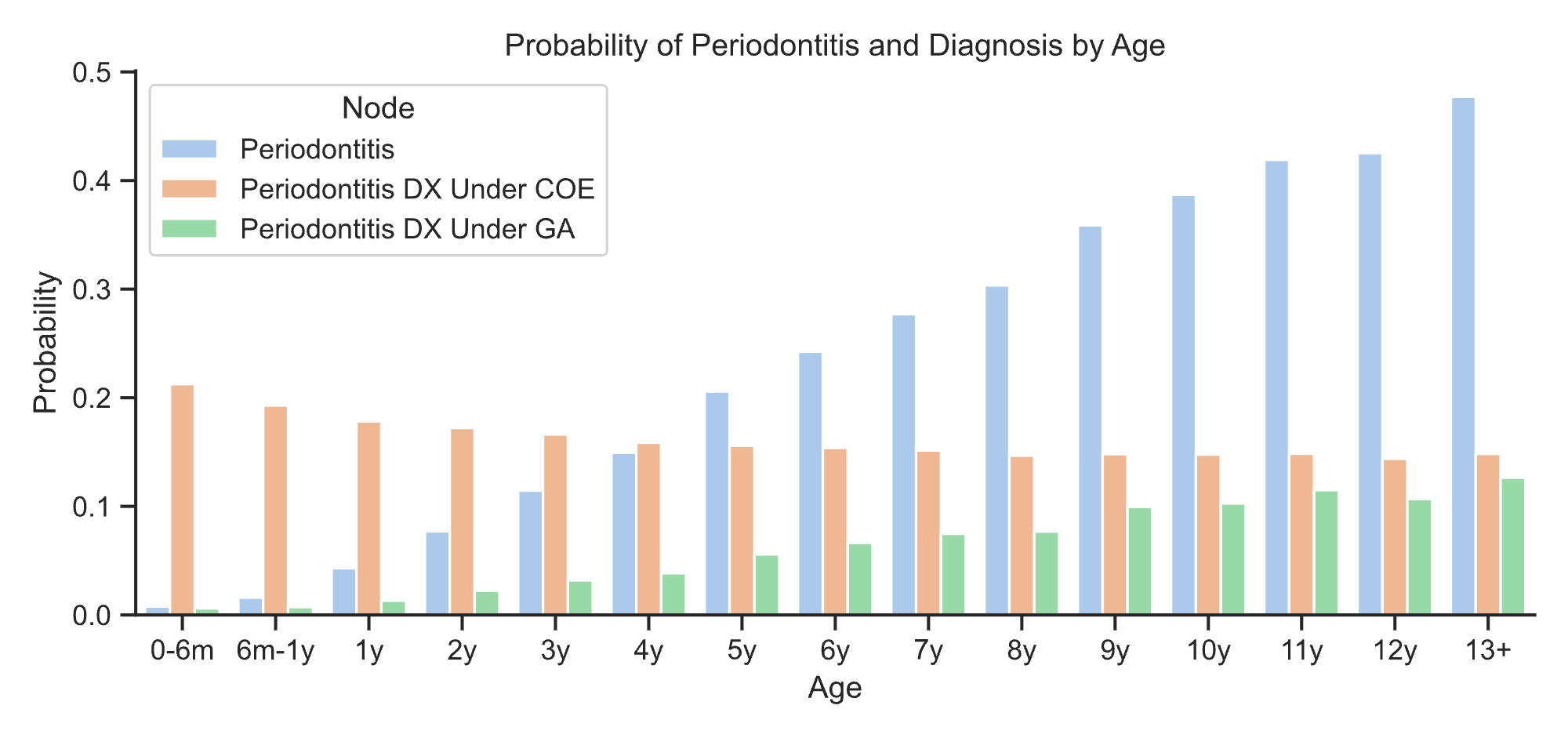

Supplement: Supplementary file 1 [file Data_Sheet_1.docx]
